# Cell proliferation fate mapping reveals regional cardiomyocyte cell-cycle activity in subendocardial muscle of left ventricle

**DOI:** 10.1038/s41467-021-25933-5

**Published:** 2021-10-01

**Authors:** Xiuxiu Liu, Wenjuan Pu, Lingjuan He, Yan Li, Huan Zhao, Yi Li, Kuo Liu, Xiuzhen Huang, Wendong Weng, Qing-Dong Wang, Linghong Shen, Tao Zhong, Kun Sun, Reza Ardehali, Ben He, Bin Zhou

**Affiliations:** 1 State Key Laboratory of Cell Biology, Shanghai Institute of Biochemistry and Cell Biology, Center for Excellence in Molecular Cell Science, Chinese Academy of Sciences, University of Chinese Academy of Sciences, Shanghai, 200031 China; 2 School of Life Science, Hangzhou Institute for Advanced Study, University of Chinese Academy of Sciences, Hangzhou, 310024 China; 3 School of Life Science and Technology, ShanghaiTech University, Shanghai, 201210 China; 4 Bioscience Cardiovascular, Research and Early Development, Cardiovascular, Renal and Metabolism (CVRM), BioPharmaceuticals R&D, AstraZeneca, Gothenburg, Sweden; 5 Department of Cardiology, Shanghai Chest Hospital, Shanghai Jiaotong University, Shanghai, China; 6 School of Life Science, East China Normal University, Shanghai, China; 7 Department of Pediatric Cardiology, Xinhua Hospital, Shanghai Jiao Tong University School of Medicine, 200092 Shanghai, China; 8 Cardiology, UCLA David Geffen School of Medicine, 675 Charles E. Young South, Los Angeles, CA 90095-1760 USA

**Keywords:** Cell proliferation, Self-renewal, Cardiac regeneration

## Abstract

Cardiac regeneration involves the generation of new cardiomyocytes from cycling cardiomyocytes. Understanding cell-cycle activity of pre-existing cardiomyocytes provides valuable information to heart repair and regeneration. However, the anatomical locations and in situ dynamics of cycling cardiomyocytes remain unclear. Here we develop a genetic approach for a temporally seamless recording of cardiomyocyte-specific cell-cycle activity in vivo. We find that the majority of cycling cardiomyocytes are positioned in the subendocardial muscle of the left ventricle, especially in the papillary muscles. Clonal analysis revealed that a subset of cycling cardiomyocytes have undergone cell division. Myocardial infarction and cardiac pressure overload induce regional patterns of cycling cardiomyocytes. Mechanistically, cardiomyocyte cell cycle activity requires the Hippo pathway effector YAP. These genetic fate-mapping studies advance our basic understanding of cardiomyocyte cell cycle activity and generation in cardiac homeostasis, repair, and regeneration.

## Abstract (web summary)

The adult mammalian heart exhibits stubbornly low levels of cardiomyocyte proliferation, leading to high morbidity after injury or heart attack. Here the authors develop an approach for tracking cardiomyocyte cell cycling and show that the majority are located adjacent to the endocardium.

## Introduction

Excessive loss of cardiomyocytes—the essential contractile muscle cells and building blocks of beating heart—can cause heart failure, one of the leading causes of death worldwide^1^. Unraveling the endogenous sources of new cardiomyocytes could enable major advances in cardiac repair and regeneration^2^. Previous cardiomyocyte lineage tracing studies and isotope incorporation assays have revealed that new cardiomyocytes are generated through self-proliferation rather than via stem cells^3–7^. While these elegant studies have documented cardiomyocyte renewal, the specific regions where new cardiomyocytes are generated within the adult heart are unclear. Despite previous studies investigating cardiomyocyte proliferation, an enduring question remains as to whether new cardiomyocytes are generated stochastically and distributed evenly throughout the heart or if newly generated cardiomyocytes are more enriched in specific anatomical locations. Long-term monitoring of in situ cardiomyocyte-specific proliferation in the adult heart remains technically challenging. First, immunostaining for proliferation marker(s) in heart sections provides only a single snapshot of cardiomyocyte proliferation, rather than a continuous view of changes over time. It is, therefore, technically challenging to detect very rare cycling cardiomyocytes on tissue sections of a normal heart. Second, tissue staining for cardiomyocyte proliferation is significantly confounded by the proliferation of non-myocytes. As the majority of proliferating cells in the adult heart are not cardiomyocytes^4,5^, in situ analysis of cardiomyocyte proliferation may have been influenced by major signal-to-noise-ratio issue for distinguishing true proliferating cardiomyocyte signals from all other proliferating cell signals in the tissue sections. Furthermore, while cardiomyocytes are interspersed within multiple other cell types, methods that assess cardiomyocytes after a purification process (e.g., FACS) would be able to study proliferation of the purified cardiomyocyte population. However, isolation of cardiomyocytes entails the loss of spatial information relevant to understanding in situ cardiomyocyte renewal. The snapshot of very rare proliferating cardiomyocytes at a single time point and the fact that they are intermingled with non-cardiomyocytes remain two technical hurdles for addressing the anatomical locations and in situ dynamics of cardiomyocyte proliferation in adult mammalian hearts. Therefore, the generation of tissue-specific seamless cell proliferation recording systems for long time window would enable higher resolution for cell proliferation studies.

In this work, we developed a genetic proliferation tracer (ProTracer) to fate map cell lineage-specific proliferation in vivo. Different from previously available methods which rely on the incorporation of nucleotide analogs^8,9^, isotope analysis^4,5,10^, or staining of proliferation markers by antibody^5,11^, ProTracer is based on a tissue-specific and temporally seamless genetic fate mapping technology for monitoring of cell proliferation, enabling high resolution in the detection of the proliferation of one specific cell lineage. However, in the case of adult cardiomyocyte lineage, ProTracer based on proliferation markers not only traces cardiomyocyte proliferation, but also those cycling cardiomyocytes without cell division. This is because the widely used proliferation marker, e.g. Ki67, is expressed throughout the cell-cycle and adult mammalian cardiomyocytes can have polyploidization or nuclear division without complete cytokinesis^12,13^. Considering that many of Ki67-expressing cardiomyocytes in the adult heart do not necessarily undergo cell division, the genetic system ProTracer developed in this study records cycling cardiomyocytes that include both dividing cardiomyocytes and non-dividing cardiomyocytes. We found ProTracer provides a high spatiotemporal resolution for cardiomyocyte-specific cell-cycle activity in the adult heart during cardiac homeostasis and after injuries. By applying ProTracer, we also observed that the majority of cycling cardiomyocytes, as well as divided cardiomyocytes (~ 13% of traced cardiomyocytes) among them, are highly restricted to the subendocardial muscle of the left ventricle in adult hearts.

## Results

### Generation of ProTracer for quantification of cell-cycle activity in mouse heart

**Fig. 1:**
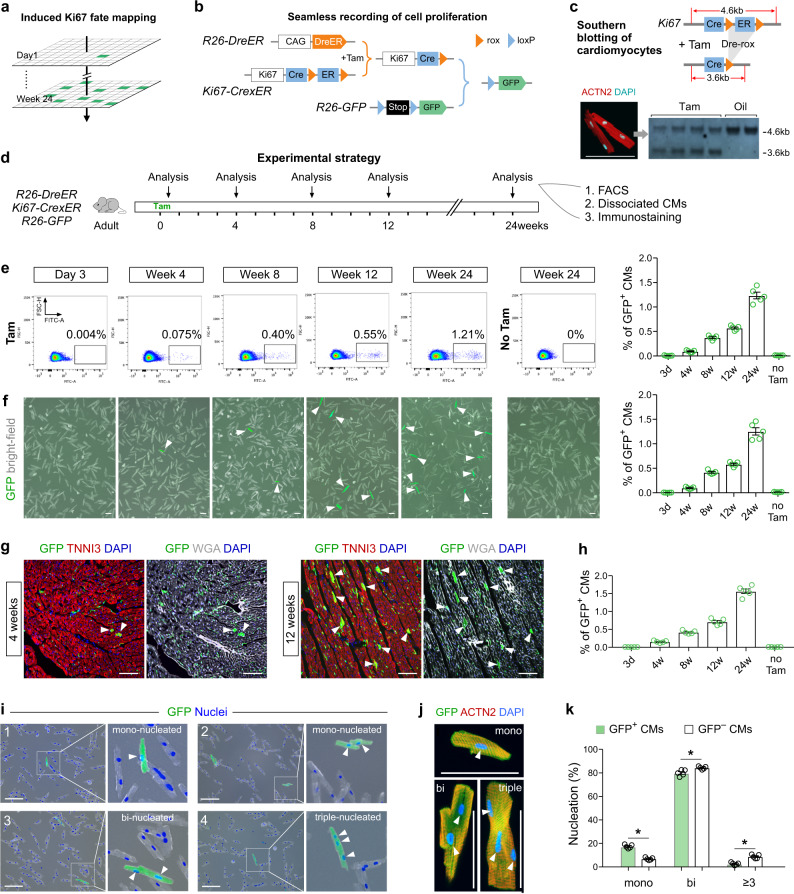
Genetic tracing of cycling cardiomyocytes using ProTracer. **a** Schematic showing recording of Ki67^+^ cells (green) from Day 1 to Week 24 in mouse tissues. **b** Strategy for the seamless recording of cell proliferation. **c** Southern blotting of isolated cardiomyocyte DNA from Tam or corn oil-treated ProTracer mice. **d** Schematic of the experimental design. **e** FACS analysis of GFP^+^ CMs and quantification of the percentage of GFP^+^ CMs (on right). Data are the mean ± s.e.m.; *n* = 5. **f** Images of dissociated CMs from mice at the indicated time points after tamoxifen treatment, and quantification of the percentage of CMs expressing GFP (on right). Arrowheads, GFP^+^ CMs. Data are mean ± s.e.m.; *n* = 5. **g** Immunostaining for GFP, TNNI3, or WGA on heart sections prepared from ProTracer mice. Arrowheads, GFP^+^ CMs. **h** Quantification of the percentage of CMs expressing GFP. Data are mean ± s.e.m.; *n* = 5. **i** Images of isolated CMs showing mononucleated, binucleated, or triple-nucleated GFP^+^ CMs. Arrowheads, nuclei. **j** Immunostaining for GFP and ACTN2 on isolated GFP^+^ CMs. Arrowheads, nuclei. **k** Quantification of the percentage of mono-, bi-, triple, or multiple nuclei-containing cells in GFP^+^ or GFP^–^ CMs. Data are the mean ± s.e.m.; *n* = 5; *P < 0.0001, *P = 0.0103, *P = 0.0001 individually using two-tailed unpaired t-test. Scale bars, 100 µm. Each image is representative of five individual biological samples.

To study cell cycle activity in mammalian hearts, we generated an approach (ProTracer) to genetically record cell cycle activity at spatiotemporal resolution (Fig. 1a, b). One of the enabling technologies of ProTracer involves the widely used cell proliferation marker Ki67^14,15^, the promoter of which has recently been exploited to drive lineage-tracing reporter in mouse brain and heart^16,17^. Since conventional *Ki67-CreER* requires continuous activation of CreER by tamoxifen, it is technically challenging to seamlessly track *Ki67* gene activity over long time window, e.g. a few months. Considering extremely rare cycling adult cardiomyocytes at a snapshot of time, and given our goal of long-term and seamless recording of cell-cycle activity in adult hearts, ProTracer was suitable because initial tamoxifen-triggered DreER-rox recombination^18^ primes continuous recording of Ki67 gene activity by *Ki67-Cre* allele thereafter^19^ (Fig. 1b). We first generated a *Ki67-Cre-rox-ER-rox* line (*Ki67-CrexER*) such that ER could be successfully excised by Dre-rox recombination (Supplementary Fig. 1). We then crossed *R26-DreER* mouse^20^, an inducible Dre driven by ubiquitous CAG promoter (also targeting cardiomyocytes), with *Ki67-CrexER* mouse, and anticipated that tamoxifen-induced DreER-rox recombination could convert *Ki67-CrexER* genotype into *Ki67-Cre* genotype, which would permanently record Ki67^+^ cells by GFP reporter^21^ thereafter (Fig. 1b). DreER-rox recombination indeed occurred in over 50% of Ki67 gene alleles in cardiomyocytes after tamoxifen treatment (Fig. 1c), indicating successful priming of the ProTracer system in the majority of cardiomyocytes.

Our first analysis of these ProTracer mice was the quantification of cardiomyocyte cell-cycle activity over a 24-weeks period in normal adult hearts. We treated tamoxifen on adult mice at age of 8–10 weeks old (set as 0 week as a start). We collected hearts from the ProTracer mice on day 3 and week 4, 8, 12, and 24 after tamoxifen priming on these adult mice (Fig. 1d). We first performed flow cytometric analysis on the dissociated cardiomyocytes from digested hearts (Supplementary Fig. 2). FACS analysis of cardiomyocytes revealed the cumulative instances of cell-cycle activity occurring over time: the proportion of GFP^+^ cardiomyocytes increased from Week 4 to Week 24, eventually reaching 1.23 ± 0.068% (Fig. 1e). Considering our labeling efficiency, this amount of cell-cycling cardiomyocytes (~ 5% per year calculated from 1.23% in 6 months with ~ 50% priming efficiency) was consistent with previous isotope incorporation study reporting a yearly rate of cardiomyocyte DNA replication of 4.4%^5^. This trend was confirmed by manually counting of dissociated cardiomyocytes (Fig. 1f) as well as by immunostaining of heart sections with antibodies against GFP, the cardiomyocyte marker TNNI3, and the cardiomyocyte membrane marker WGA (Fig. 1g, h and Supplementary Fig. 3). Note that we detected no GFP^+^ cardiomyocytes in non-Tam priming control in any of the above analyses (Fig. 1e–h and Supplementary Fig. 3). We also dissociated cardiomyocytes for further analysis of their nucleation (Fig. 1i, j), and found that the percentage of mono-nuclear cardiomyocytes in the GFP^+^ population was significantly higher than that of the GFP^–^ population (Fig. 1k). The above genetic results provided detailed quantification of cardiomyocyte cell-cycle activity from 3 days to 6 months’ duration in adult mammalian hearts during homeostasis.

### Highly regional cycling cardiomyocytes revealed by cardiomyocyte-specific ProTracer

**Fig. 2:**
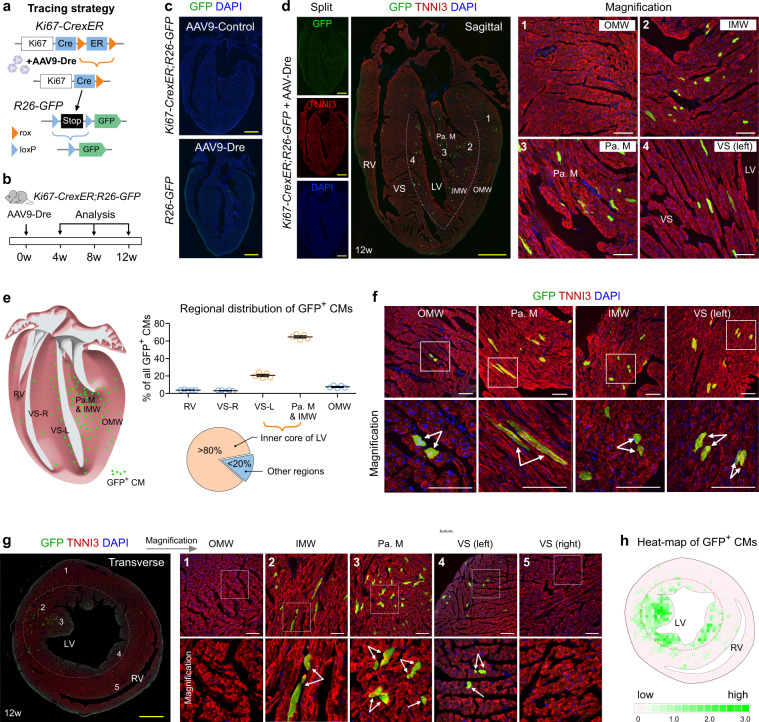
Cardiomyocyte-specific ProTracer identifies highly regional cardiomyocyte cell-cycle activity in adult hearts. **a** Schematic showing AAV9-Dre-induced tracing of cardiomyocyte cell-cycle activity. **b** Schematic illustrating the experimental design. **c** Immunostaining for GFP on heart sections prepared from *Ki67-CrexER*;*R26-GFP* mice treated with AAV9-control or *R26-GFP* mice treated with AAV9-Dre. **d** Immunostaining for GFP and TNNI3 on heart sections collected from *Ki67-CrexER*;*R26-GFP* mice treated with AAV9-Dre. Numbered regions (1,2,3,4) are magnified on the right. OMW outer myocardial wall, IMW inner myocardial wall, Pa. M papillary muscle, VS ventricular septum, RV right ventricle, LV left ventricle. **e** Illustration of cycling CMs (GFP) in the adult heart in 12 weeks. Quantification of the distribution of GFP^+^ CMs in different regions of the ventricles. Data are the mean ± s.e.m.; *n* = 5. **f** Representative images showing GFP^+^ CMs (arrows) in different regions of hearts. **g** Immunostaining for GFP and TNNI3 in transverse heart sections. The dotted line demarcates the inner core region of the LV that harbors the majority of the GFP^+^ cardiomyocytes in the ventricles. Numbered regions (1–5) are magnified on the right. Arrows, GFP^+^ cardiomyocytes. **h** Heat-map of GFP^+^ cardiomyocytes (CM) in transverse heart sections. The green color intensity indicates the average number of GFP^+^ CMs in each 0.0256 mm^2^ square from five heart sections. The red dotted line demarcates the inner core region of the LV. Scale bars, yellow, 1 mm; white, 100 µm. Each image is representative of five individual biological samples.

In order to clearly visualize the cumulative instances of cell-cycle activity specifically in cardiomyocytes, i.e., without the aforementioned signal interference from non-myocyte cell lineages, we next employed a cardiomyocyte-targeting virus to uniquely prime the ProTracer recording system in cardiomyocytes (Fig. 2a). Specifically, we treated *Ki67-CrexER;R26-GFP* mice of 8–10 weeks old (set as 0w) with AAV9-Dre, which is known to have strong cardiomyocyte tropism^22^, and collected heart tissues for analysis after 4, 8, and 12 weeks (Fig. 2b). For technical controls, analysis of the AAV9-control-treated *Ki67-CrexER;R26-GFP* mice or AAV9-Dre-treated *R26-GFP* mice showed no GFP signals in heart tissues (Fig. 2c). In AAV9-Dre treated *Ki67-CrexER;R26-GFP* mice, GFP was specifically expressed in TNNI3^+^ cardiomyocytes (Fig. 2d). To ensure accurate quantification of cardiomyocytes only, GFP^+^TNNI3^+^ double-positive cells were examined. Fluorescent imaging of whole-heart sections revealed highly enriched GFP^+^ cardiomyocytes in the papillary muscles, that serve as the anchor for the mitral valve (Fig. 2d). In addition to the papillary muscles, the inner region of the left ventricle (LV) neighboring the endocardium had significantly more GFP^+^ cardiomyocytes than that of the outer myocardial wall (Fig. 2d). Overall, we observed significantly more GFP^+^ cardiomyocytes in the free wall of left ventricle (LV) than that of the right ventricle (RV). Within the interventricular septum, GFP^+^ cardiomyocytes were preferentially localized to the left side and near the left ventricular cavity (Fig. 2d and Supplementary Fig. 4). Very few GFP^+^ cardiomyocytes were observed in the right ventricular wall or on the right side of the interventricular septum (Fig. 2d and Supplementary Fig. 4). Quantitatively, we noted more than 80% of GFP^+^ cardiomyocytes resided in the inner core of the left ventricle (Fig. 2d, e). Magnified images of heart sections stained with GFP and cardiomyocyte marker TNNI3 showed that these GFP^+^ signals were colocalized with TNNI3 (arrows) in different regions of adult hearts (Fig. 2f), demonstrating the cardiomyocyte-specific recording of cell-cycle activity. Since non-myocytes minimally fuse with cardiomyocytes^7,23^, it is unlikely most of these detected GFP^+^ cardiomyocytes are generated by cell fusion from proliferating non-cardiomyocytes un-specifically targeted by AAV9-Dre.

The above data from coronal sections of hearts (four-chamber view) showed the enrichment of GFP^+^ cardiomyocytes in the subendocardial muscle of the LV, particularly the papillary muscle. To view this through another anatomic angle, we next performed transverse heart sections to study the distribution of cardiomyocyte cell-cycle activity in adult hearts. Immunostaining data from transverse heart sections also showed the majority of GFP^+^ cardiomyocytes were mainly restricted to the inner core of the left ventricle, particularly the papillary muscle and the left side of the ventricular septum (Fig. 2g). Magnification of different regions of transverse heart sections revealed that these GFP^+^ cells were TNNI3^+^ cardiomyocytes (Fig. 2g). Heat-map of GFP^+^ cell frequency from the similar level of transverse sections from five individual mice quantitatively showed highly enriched GFP^+^ cardiomyocytes in the papillary muscle and the left side of ventricular septum facing LV chamber (Fig. 2h). These data demonstrated that adult cardiomyocyte cell-cycle activity is not randomly distributed in the heart, but exhibited highly regional patterning.

### A subset of GFP^+^ cardiomyocytes labeled by ProTracer have undergone cell division

**Fig. 3:**
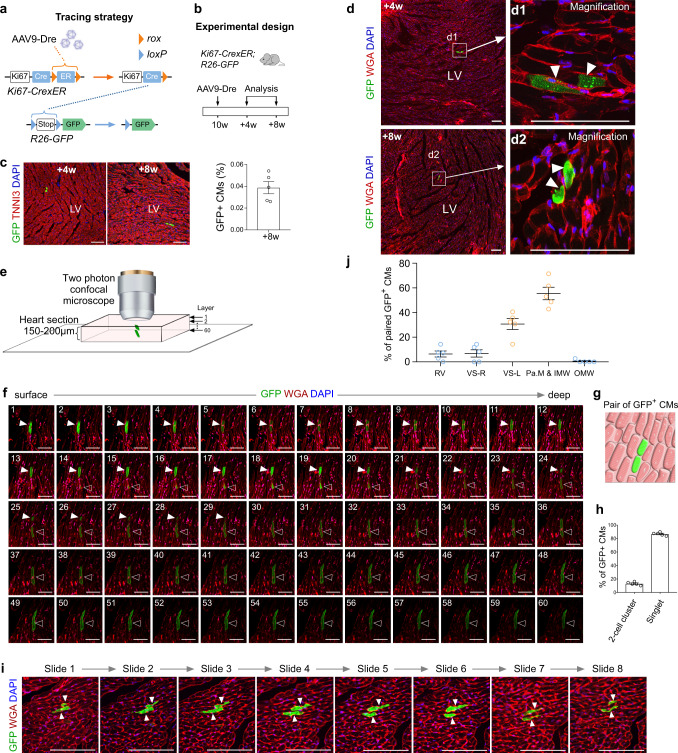
A subset of Ki67^+^ cardiomyocytes labeled by ProTracer have undergone cytokinesis. **a** Schematic showing strategy of labeling Ki67^+^ cardiomyocytes by AAV9-Dre injection into *Ki67-CrexER*;*R26-GFP* mice. **b** Schematic showing the experimental design. A low dosage of AAV9-Dre was injected into mice. **c** Immunostaining for GFP and TNNI3 on heart sections reveals sparse labeling of cardiomyocytes (CMs) and quantification of the percentage of CMs expressing GFP. **d** Immunostaining for GFP and WGA on 10 µm heart sections reveals sparsely labeled CMs (< 0.1%) on heart sections collected at 4 or 8 weeks after AAV injection. Arrowheads, sparse pair of GFP^+^ CMs. **e**, **f** Confocal microscopy of thick heart sections (150–200 µm) reveals two neighboring GFP^+^ CMs (arrowheads). Each stacked image is composed of ~ 60 layers of scanned signals of heart sections stained with GFP and WGA. **g** Cartoon image showing a pair of two neighboring GFP^+^ CMs. **h** Quantification of the percentage of GFP^+^ CMs contributed from 2-cell cluster or single-cell cluster (singlet). Data are the mean ± SEM; *n* = 5. **i** Immunosta**i**ning for GFP and WGA on consecutive tissue sections (serial tissue slide 1 to slide 8, each 10 µm) revealed a pair of neighboring CMs (arrowheads). **j** Quantification of the distribution of GFP^+^ 2-cell clusters in different regions of the ventricles. Data are the mean ± s.e.m.; *n* = 5. Scale bars: 100 µm. Each image is representative of five individual biological samples.

As Ki67 is expressed in DNA replicating or nuclear dividing cells that have not completed cytokinesis, the above quantification results only represented the cell-cycle activity. The cardiomyocytes traced by ProTracer not only include newly generated ones but also those who have initiated cell cycle without completion. Nevertheless, the benefit of our genetic approach and its attendant high signal-to-background resolution is the ability to permanently record those neighboring pairs of GFP^+^ cardiomyocytes when they divide, given that Ki67^+^ cardiomyocytes could be sparsely labeled by ProTracer. Therefore, we performed a new experiment by reduced AAV9-Dre induction that yielded very sparse GFP labeling of Ki67^+^ cardiomyocytes (Fig. 3a). We collected heart samples from *Ki67-CrexER;R26-GFP* mice at 4 or 8 weeks after AAV9-Dre treatment (Fig. 3b). Quantification analysis on the heart sections collected at 8 weeks after AAV9-Dre treatment showed that 0.039 ± 0.0056% of TNNI3^+^ cardiomyocytes in the *Ki67-CrexER;R26-GFP* heart were GFP positive (Fig. 3c), indicating the sparse labeling of Ki67^+^ cardiomyocytes by ProTracer system. Considering the rarity of GFP^+^ cycling cardiomyocytes (< 0.05%), the occurrence of two neighboring GFP^+^ cardiomyocytes can be assumed to represent two post-cytokinesis daughter cells. Immunostaining for GFP and WGA (for cardiomyocyte membrane labeling) on heart sections showed two neighboring cardiomyocytes expressing GFP (Fig. 3d), indicating post-cytokinesis. Z-stack confocal imaging of thick heart sections (150–200 µm) confirmed these adjacent GFP^+^ cardiomyocytes were two individual cardiomyocytes (Fig. 3e, f), indicating a subset of cycling cardiomyocytes have undergone cell division or cytokinesis in the adult heart during homeostasis (Fig. 3g). Quantitatively, 13.19 ± 1.07% of GFP^+^ cardiomyocytes were found in pairs of neighboring cardiomyocytes (2-cell cluster); while ~ 86% of GFP^+^ cardiomyocytes were detected as singlet (Fig. 3h). To circumvent the weak staining on thick tissue sections (e.g. deep regions), we collected series of consecutive heart sections at 10 µm each. Immunostaining for GFP and WGA on these sister sections again revealed pairs of two neighboring GFP^+^ cardiomyocytes (Fig. 3i). Of note, the majority of these paired cardiomyocytes (> 80%) were detected in the inner core of left ventricle that includes papillary muscle and inner myocardial wall (Fig. 3j), indicating regional cardiomyocyte proliferation in adult hearts during cardiac homeostasis. Taken together, these data provided genetic evidence that a subset of Ki67-expressing cardiomyocytes (e.g. those paired cardiomyocytes) have indeed undergone proliferation or cytokinesis, therefore giving rise to additionally new cardiomyocytes in the adult heart.

### *Tnnt2-DreER* primed ProTracer system revealed regional cardiomyocyte cell-cycle activity

**Fig. 4:**
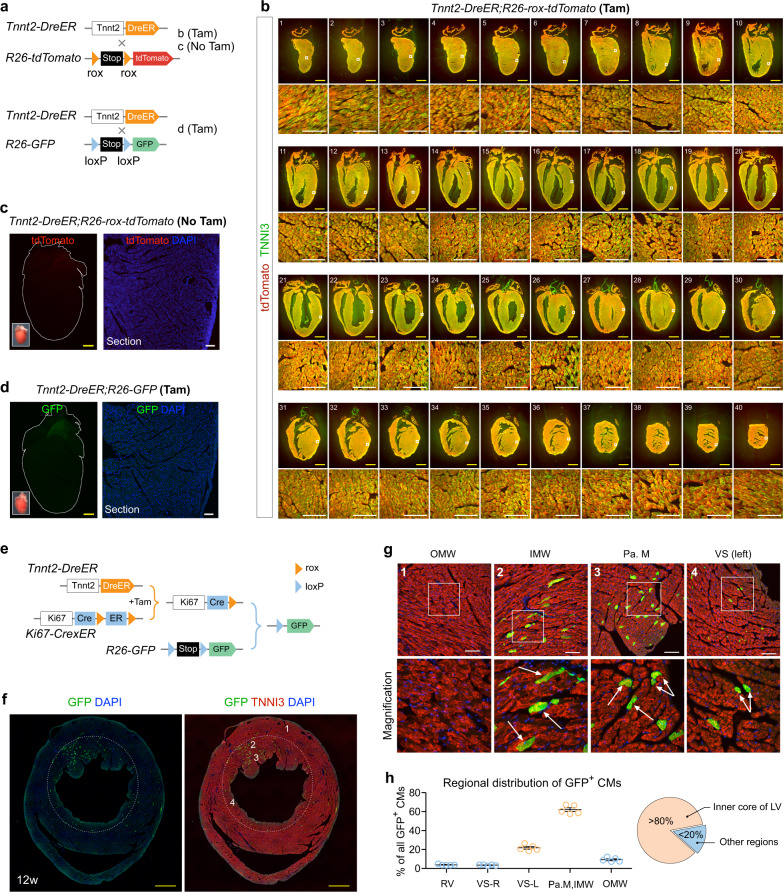
*Tnnt2-DreER* triggered ProTracer recording system reveals highly regional cardiomyocyte cell-cycle activity. **a** Schematic showing crossing of *Tnnt2-DreER* with rox or loxP reporters. **b** Immunostaining for tdTomato and TNNI3 on 1–40 serial heart sections collected from *Tnnt2-DreER*;*R26-rox-tdTomato* mice treated with tamoxifen (Tam). Boxed regions are magnified in the lower panels. **c** Whole-mount and sectional fluorescent images of hearts collected from *Tnnt2-DreER*;*R26-rox-tdTomato* mice without tamoxifen treatment (No Tam). **d** Whole-mount and sectional fluorescent images of hearts collected from *Tnnt2-DreER*;*R26-GFP* mice treated with tamoxifen (Tam). **e** Schematic showing a strategy using *Tnnt2-DreER* to prime ProTracer system for recording of cardiomyocyte cell-cycle activity. **f**, **g** Immunostaining for GFP and TNNI3 on *Tnnt2-DreER*;*Ki67-CrexER*;*R26-GFP* heart sections. Numbered regions (1,2,3,4) are magnified on the (**g**) panels. Arrows, GFP^+^ cardiomyocytes. OMW outer myocardial wall, IMW inner myocardial wall, Pa. M papillary muscle, VS ventricular septum. **h** Quantification of the distribution of GFP^+^ CMs in different regions of hearts. Data are mean ± s.e.m.; *n* = 5. Scale bars: yellow, 1 mm; white, 100 µm. Each figure is representative of five individual biological samples.

One caveat of using AAV9-Dre to prime ProTracer system in cardiomyocytes is the potential preferential infection of specific regions, e.g. possibly in the papillary muscle, therefore, leading to the highly enriched GFP^+^ cardiomyocytes pattern in that regions. To independently study the cell-cycle activity of cardiomyocytes throughout the adult heart, we generated an alternative strategy for priming cardiomyocyte-specific ProTracer (Fig. 4a). We generated cardiomyocyte-specific DreER, *Tnnt2-DreER*, to prime the ProTracer system using tamoxifen treatment. We first characterize whether *Tnnt2-DreER* targets cardiomyocytes unbiasedly throughout the adult heart. By crossing with rox reporter *R26-rox-tdTomato*^24^, *Tnnt2-DreER* efficiently and unbiasedly targeted cardiomyocytes in different regions of adult hearts after tamoxifen treatment (Fig. 4b). As a control, *Tnnt2-DreER* did not recombine rox flanked allele without tamoxifen treatment (Fig. 4c). We did not find any tamoxifen-induced Dre-loxP recombination when *Tnnt2-DreER* was crossed with loxP reporter *R26-GFP* (Fig. 4d), indicating that robust *Tnnt2-DreER* driver would not directly activate *R26-GFP* reporter.

We next crossed *Tnnt2-DreER* with *Ki67-CrexER;R26-GFP* mice, and anticipated that tamoxifen-induced DreER-rox recombination would switch *Ki67-CrexER* into *Ki67-Cre* genotype specifically in Tnnt2^+^ cardiomyocytes (Fig. 4e). We treated tamoxifen on *Tnnt2-DreER*;*Ki67-CrexER;R26-GFP* mice at 8–10 weeks of age, and collected hearts 12 weeks later. Fluorescent imaging of heart sections stained for GFP and TNNI3 revealed highly restricted pattern of GFP^+^ cardiomyocyte to particular regions such as the inner core of the LV (Fig. 4f). Magnified images of different regions of heart sections confirmed that GFP^+^TNNI3^+^ cardiomyocytes were highly enriched in the papillary muscles and its abutting region of the inner myocardial wall of the LV (Fig. 4g). Quantitatively, over 80% of GFP^+^TNNI3^+^ cardiomyocytes were restricted in the inner core of the LV, with < 20% in the other regions of the ventricles (Fig. 4h), suggesting highly regional cardiomyocyte cell cycle activity. As a technical control, *Ki67-CrexER;R26-GFP* mice were treated with tamoxifen at age of 8–10 weeks (set as 0w) and hearts were collected at 12 weeks after tamoxifen treatment. In heart sections, we could hardly observe any GFP^+^TNNI3^+^ cardiomyocytes (about 1 GFP^+^ cardiomyocyte every section, Supplementary Fig. 5). Very few GFP^+^ non-cardiomyocyte cell lineages were observed (about 30 GFP^+^ endothelial cells and 2 fibroblasts every section, Supplementary Fig. 5), which was due to CrexER activity induced by tamoxifen in *Ki67-CrexER;R26-GFP* mice. Taken together, genetic fate mapping results by *Tnnt2-DreER* primed ProTracer system revealed regional cycling cardiomyocytes in the adult heart.

### Ccna2-based ProTracer confirms the pattern of regional cycling cardiomyocytes

**Fig. 5:**
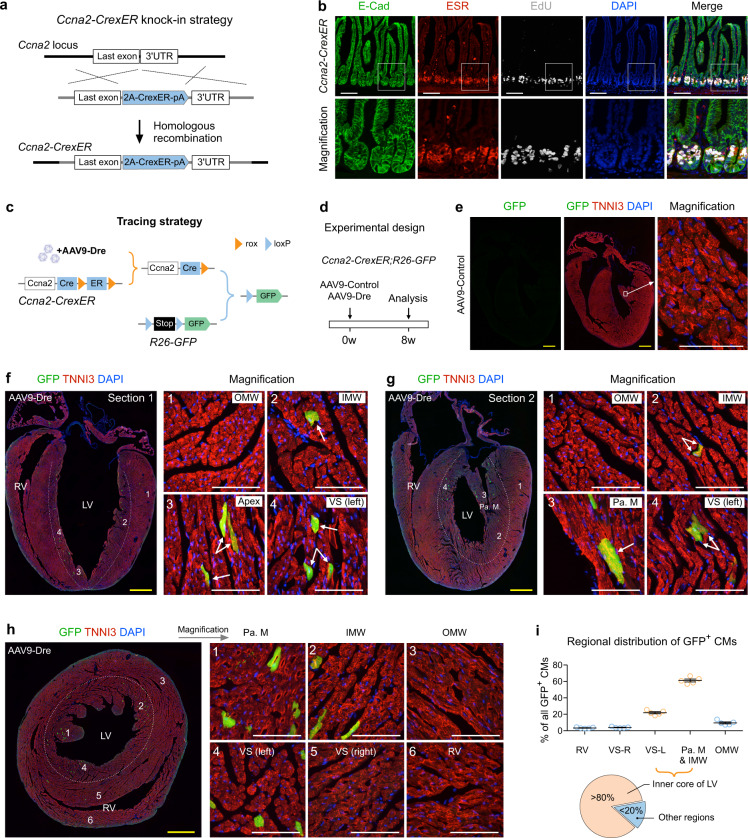
Cyclin A2 (*Ccna2*) based ProTracer reveals a highly regional pattern of cardiomyocyte cell-cycle activity. **a** Schematic showing generation of a *Ccna2-CrexER* knock-in allele. **b** Immunostaining for E-Cad, ESR, and EdU on intestinal sections from adult (10 weeks old) *Ccna2-CrexER* mice. Mice received intraperitoneal EdU injection and were sacrificed 3 h afterward for analysis. **c** Schematic showing AAV9-Dre induced genetic tracing of Ccna2^+^ cardiomyocytes. **d** Schematic of the experimental design. **e** Immunostaining for GFP and TNNI3 on heart sections prepared from AAV9-control-treated mice. **f**, **g** Immunostaining for GFP and TNNI3 on heart sections from AAV9-Dre treated mice. Numbered regions (1,2,3,4) are magnified on the right. Arrows, GFP^+^ cardiomyocytes. **h** Immunostaining for GFP and TNNI3 in transverse heart sections. The dotted line demarcates the inner core region of the LV that harbors the majority of GFP^+^ cardiomyocytes. Numbered regions (1–6) are magnified on the right. **i** Quantification of the distribution of GFP^+^ CMs in different regions of the ventricles. Data are the mean ± s.e.m.; *n* = 5. Scale bars: yellow, 1 mm; white, 100 µm. Each image is representative of five individual biological samples.

To independently corroborate the above observation that cardiomyocyte cell-cycling activity is highly regional, we generated another ProTracer strategy based on another widely used cell-cycle marker, Cyclin A2 (encoded by *Ccna2* gene), which has been previously used as cardiomyocyte cell cycle indicator^25^. Similar to the *Ki67*-ProTracer mice, we generated the required *Ccna2-CrexER* mouse allele by inserting CrexER into the 3' UTR of the *Ccna2* gene using the self-cleaved peptide P2A (Fig. 5a). In *Ccna2-CrexER* adult mice, the CrexER expression pattern accurately mirrored cell-cycling regions in the small intestine (Fig. 5b), indicating Ccna2 gene is specifically expressed in cycling cells. We next treated *Ccna2-CrexER;R26-GFP* mice of 8–10 weeks old (set as 0w) with AAV9-Dre, and anticipated that Dre-rox recombination in cardiomyocytes would switch *Ccna2-CrexER* genotype into the *Ccna2-Cre* genotype, thus priming the ProTracer system for continuous recording of cardiomyocyte cell-cycle activity (Fig. 5c, d). We did not observe any GFP^+^ cardiomyocytes in AAV9-Control treated mice (Fig. 5e). Fluorescent imaging of whole-heart sections of AAV9-Dre treated mice showed a highly restricted pattern of GFP^+^ cardiomyocytes in the papillary muscle and its abutting region of the inner myocardial wall of the LV (Fig. 5f, g). Moreover, similar to the *Ki67*-ProTracer hearts, we observed pronounced left-side enrichment for GFP^+^ cardiomyocytes in the ventricular septum of the *Ccna2*-ProTracer hearts (Fig. 5f, g). This highly regional cardiomyocyte cell-cycle activity in adult hearts was also confirmed with transverse sections of *Ccna2*-ProTracer hearts (Fig. 5h). Quantitatively, over 80% of GFP^+^ cardiomyocytes in the ventricles were restricted to the inner core of the left ventricle (Fig. 5i), findings consistent with the *Ki67*-ProTracer heart data. The above data revealed highly regional cycling cardiomyocytes in the adult heart during homeostasis.

### Regional Ki67 expression and EdU incorporation in cardiomyocytes of adult hearts

**Fig. 6:**
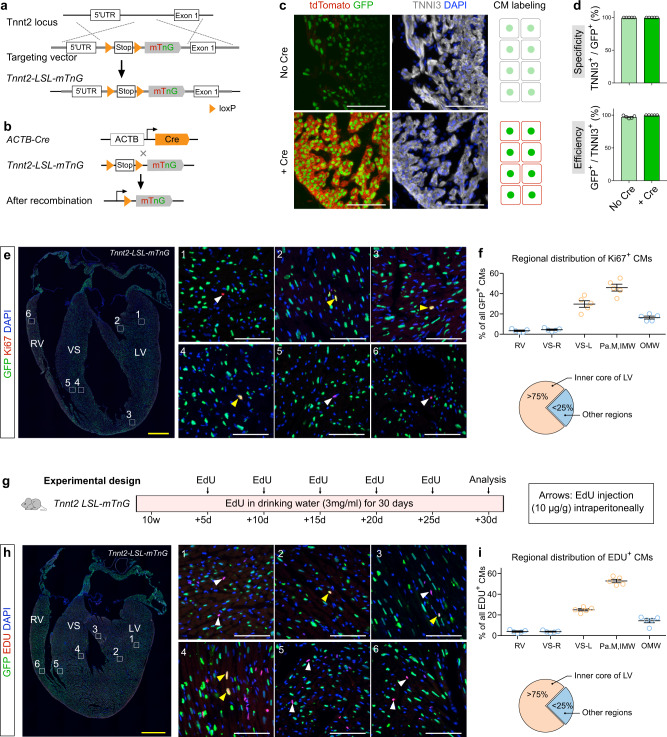
Cardiomyocyte-specific Ki67 protein expression and EdU incorporation are more enriched in inner core of left ventricle. **a** Schematic figure showing generation of Tnnt2-loxP-Stop-loxP-membrane tdTomato-2A-nuclear GFP (*Tnnt2-LSL-mTnG*) mouse line. **b** Strategy of crossing Cre line with *Tnnt2-LSL-mTnG* line. **c** Immunostaining for GFP, tdTomato, and TNNI3 on heart tissue sections of E13.5 *Tnnt2-LSL-mTnG* embryos containing Cre or no Cre. Cartoon shows labeling of cardiomyocytes (CM) in no Cre or Cre embryos. **d** Quantification of the GFP labeling specificity and efficiency in Tnnt2-LSL-mTnG containing Cre or no Cre embryos. Data are mean ± s.e.m.; *n* = 5. **e** Immunostaining for Ki67 and GFP on *Tnnt2-LSL-mTnG* heart sections (10 weeks). Boxed regions (1–6) are magnified on the right panel. Yellow arrowheads, nGFP^+^Ki67^+^ CMs; white arrowheads, nGFP^–^Ki67^+^ nuclei. **f** Quantification of the distribution of nGFP^+^Ki67^+^ CMs in different regions of hearts. Data are mean ± s.e.m.; *n* = 5. **g** Schematic figure showing the experimental design. **h** Immunostaining for GFP and EdU on heart sections (14 weeks). Boxed regions (1–6) are magnified on the right panel. Yellow arrowheads, nGFP^+^EdU^+^ CMs; white arrowheads, nGFP^–^EdU^+^ nuclei. **i** Quantification of the distribution of nGFP^+^EdU^+^ CMs in different regions of hearts. Data are mean ± s.e.m.; *n* = 5. Each image is representative of five individual biological samples. Scale bars: yellow, 1 mm; white, 100 µm.

To independently address whether cell-cycling cardiomyocytes are more enriched in some myocardial regions, we performed conventional experiments by immunostaining for cell-cycling or proliferation markers. To specifically quantify the cell-cycling cardiomyocytes, we first generated cardiomyocyte-specific reporter *Tnnt2-loxP-Stop-loxP*-*membrane tdTomato*-*nuclear GFP* mouse line, *Tnnt2-LSL-mTnG* (Fig. 6a). Crossing of this reporter with *ACTB-Cre* would remove Stop cassette, thus enabling specific expression of membrane tdTomato (mT) and nuclei GFP (nG or nGFP) in cardiomyocytes (Fig. 6b). We found that weak nGFP was expressed in cardiomyocytes of *Tnnt2-LSL-mTnG* mice without Cre (No Cre group) (Fig. 6c and Supplementary Fig. 6). This might be due to the leakiness of mTnG, and nucleus-restricted GFP was more readily detection, while membrane-diffused tdTomato was not readily visualized. In the presence of Cre (+Cre group), strong nG expression, as well as mT expression, were detected specifically in cardiomyocytes (Fig. 6c and Supplementary Fig. 6). Quantification of weak or strong nGFP expression in No Cre or Cre group, respectively, revealed the high specificity and efficiency of cardiomyocyte labeling in both groups (Fig. 6d).

While nGFP but not mT was leaky in *Tnnt2-LSL-mTnG* mice for unknown reason, most cardiomyocytes were specifically and efficiently labeled by this leaky nGFP. We thus took advantage of this leakiness of nG for nuclei quantification of cell-cycling cardiomyocytes by Ki67 staining or EdU incorporating. Immunostaining for GFP and Ki67 on whole-heart sections revealed extremely rare Ki67^+^ CMs in the adult heart, ranging from 0.0052% to 0.0083% in five examined samples (Fig. 6e). In spite of this rarity, we quantified the distribution of nGFP^+^Ki67^+^ cardiomyocytes in different regions of ventricles in all collected sections of adult hearts (~ 500 sections per heart, *n* = 5). Quantification data revealed Ki67^+^ cardiomyocytes were more restricted in the inner core of the left ventricle that includes papillary muscle, inner myocardial wall, and the left side of the ventricular septum (Fig. 6f). Additionally, we treated *Tnnt2-LSL-mTnG* mice with EdU and stained heart sections with GFP and EdU at 30 days after treatment (Fig. 6g). Similarly, very rare nGFP^+^EdU^+^ cardiomyocytes were detected in the heart sections, accounting for 0.503% of cardiomyocytes in the adult heart (Fig. 6h). Quantification data revealed that over 75% of EdU^+^ cardiomyocyte nuclei were distributed in the inner core of the left ventricle (Fig. 6i). These data, without relying on genetic lineage tracing, independently showed that cell-cycling cardiomyocytes are more restricted to the regional myocardium of the left ventricle wall during cardiac homeostasis.

To experimentally address whether *Ki67-Cre* traced cardiomyocytes have indeed undergone cell-cycle activity, we used the widely accepted EdU incorporation assay to determine if these labeled cardiomyocytes also incorporate EdU (Supplementary Fig. 7). For 30 days of genetic tracing, we treated ProTracer (*R26-DreER*;*Ki67-CrexER*;*R26-GFP*) mice with EdU and then collected tissue samples for staining of GFP, EdU, and TNNI3. We found that the majority of GFP^+^ cardiomyocytes were EdU^+^ (Supplementary Fig. 7a, b, e). Additionally, we treated two different cardiomyocyte-specific ProTracer (*Tnnt2-DreER*;*Ki67-CrexER*;*R26-RSR-LSL-tdTomato* or *Tnnt2-DreER*;*Ccna2-CrexER*;*R26-RSR-LSL-tdTomato*) with EdU for 60 days to further validate the above result (Supplementary Fig. 7c). Immunostaining on tissue sections revealed that the majority of tdTomato^+^ cardiomyocytes were EdU^+^ (Supplementary Fig. 7d). Quantification data from the above three sets of genetic lineage tracing experiments showed that about 80% of the GFP^+^ or tdTomato^+^ cardiomyocytes incorporated EdU (Supplementary Fig. 7e), indicating that Ki67 or Ccna2-traced cardiomyocytes have undergone cell cycle activity.

### Increased cycling cardiomyocytes after myocardial infarction revealed by ProTracer

**Fig. 7:**
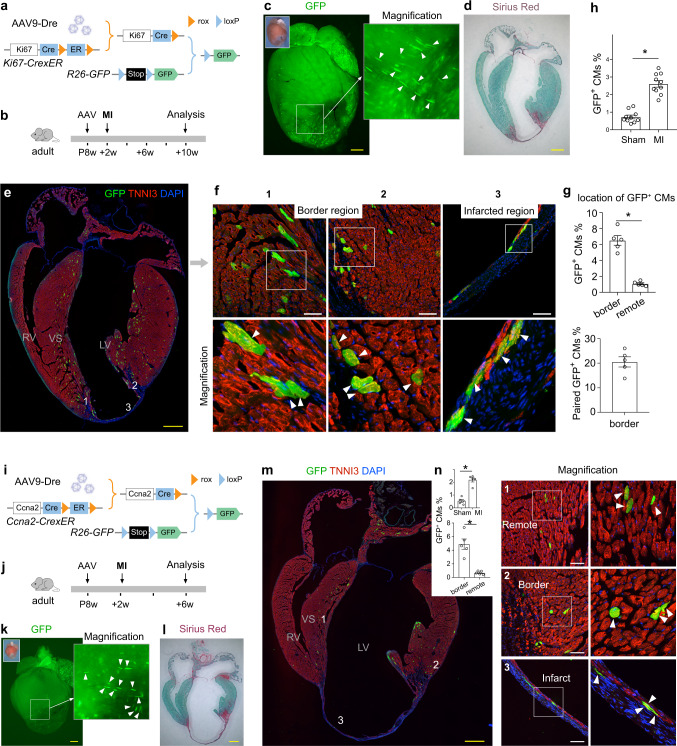
Cardiomyocyte cell-cycle activity detected by ProTracer after myocardial infarction. **a** Schematic showing Ki67-based ProTracer system. **b** Schematic showing experimental design. **c** Whole-mount GFP fluorescence of MI hearts. Magnified image shows GFP^+^ cardiomyocytes (arrowheads). **d** Sirius Red staining on MI heart sections. **e**, **f** Immunostaining for GFP and TNNI3 on MI heart sections. Regions 1, 2, 3 are magnified on the right panels. Arrowheads, GFP^+^ cardiomyocytes. **g** Quantification of GFP^+^ CM percentage in different regions of the hearts and the percentage of paired GFP^+^ cardiomyocytes in GFP^+^ cardiomyocytes adjacent the infarct. Data are mean ± s.e.m.; *n* = 5; *P < 0.0001. **h** Quantification of GFP^+^ CM percentage in Sham or MI hearts. Data are mean ± s.e.m.; *n* = 10; *P < 0.0001. **i** Schematic showing CCn2a-based ProTracer system. **j** Schematic showing experimental design. **k** Whole-mount GFP fluorescence of MI hearts. Magnified image shows GFP^+^ cardiomyocytes (arrowheads). **l** Sirius Red staining on MI heart sections. **m** Immunostaining for GFP and TNNI3 on MI heart sections. Regions 1, 2, 3 are magnified on the right panels. Arrowheads, GFP^+^ cardiomyocytes. **n** Quantification of GFP^+^ CM percentage in Sham or MI hearts and the location of GFP^+^ cardiomyocytes in different regions of the MI hearts. Data are mean ± s.e.m.; *n* = 5; *P < 0.0001, *P = 0.0006; Data were analysed by two-tailed unpaired *t*-test (g, h, n). Scale bars: yellow, 1 mm; white, 100 µm. Each image is representative of five individual biological samples.

We next applied ProTracer mice to examine cardiomyocyte cell-cycle activity after cardiac injuries. We again used *Ki67-CrexER;R26-GFP* mice and treated them with AAV9-Dre to prime ProTracer system for genetic recording of cardiomyocyte cell-cycle activity (Fig. 7a). AAV9-Dre was administered on adult mice at 8 weeks old (P8w). For cardiac injury, we performed myocardial infarction (MI) model by ligation of the left anterior descending coronary artery at 2 weeks after AAV9-Dre treatment, and collected MI hearts for analysis at 8 weeks after MI (Fig. 7b). Whole-mount fluorescence images of MI hearts showed pronounced GFP^+^ signals in the infarcted and border regions (Fig. 7c), the area of which had exhibited significant fibrosis (Fig. 7d). Immunostaining for GFP and TNNI3 on whole-heart sections revealed that regional cycling cardiomyocyte pattern still maintains, with much reduced numbers in the thin left ventricular free wall that exhibit fibrosis/scar (Fig. 7e, f). Magnified images showed that survived cardiomyocytes in the infarct regions were mainly located in the subendocardial layer, some of which expressed GFP (Fig. 7f). Quantification data showed that the percentage of GFP^+^ cardiomyocytes was significantly higher in the border region than that of remote region, indicating their response to wound healing stimuli (Fig. 7g). Quantitatively, there was a significant increase in the percentage of cardiomyocytes expressing GFP in the MI hearts compared to that in sham hearts (Fig. 7h). Our quantification data revealed that about 6.5% x 20.5% = 1.3% of the cardiomyocytes adjacent to the infarct had undergone cell proliferation, when Senyo and colleagues^5^ reported that 3.2% of the cardiomyocytes adjacent to the infarct had undergone cell proliferation. Considering our 50% priming efficiency of the ProTracer system and different methods used to evaluate cell proliferation, our results are consistent with their work (ours 1.3% x 2 = 2.6% in comparison with Richard Lee’s 3.2%). We also confirmed that the regional cardiomyocyte-cycling pattern is largely maintained after MI in *Ccna2*-based ProTracer hearts (Fig. 7i–l), demonstrating a significant increase in the proportion of GFP^+^ cardiomyocyte in MI compared with sham hearts and in border regions compared with remote region (Fig. 7m, n). Taken together, these above fate mapping results indicated that MI accelerated cardiomyocyte cell-cycle activity.

### Pressure overload induced cardiomyocyte cell-cycle activity

**Fig. 8:**
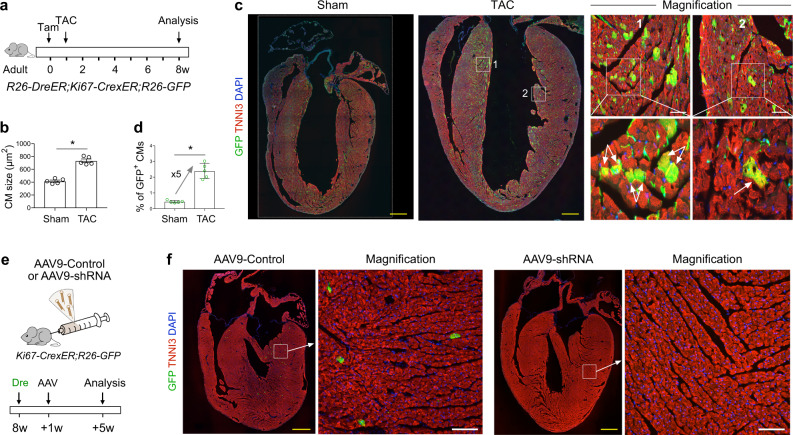
Pressure overload and Hippo pathway influence cardiomyocyte cell-cycle activity in the adult heart. **a** Schematic showing experimental design. **b** Quantification of cardiomyocyte (CM) size in sham or TAC hearts. Data are mean ± s.e.m.; *n* = 5; *P < 0.0001 when using two-tailed unpaired *t*-test. **c** Immunostaining for GFP and TNNI3 on heart sections from *R26-DreER*;*Ki67-CrexER*;*R26-GFP* mouse after Sham or TAC. Boxed regions c1 and c2 are magnified on the right panel. Arrows, GFP^+^ cardiomyoyctes. **d** Quantification of the percentage of cardiomyocyte expressing GFP. Data are mean ± s.e.m.; *n* = 5; *P < 0.0001 when using two-tailed unpaired *t*-test. **e** Schematic showing experimental design. **f** Immunostaining for GFP and TNNI3 on heart sections. Scale bars: yellow 1 mm; white 100 µm. Each image is representative of 5 individual biological samples.

Our findings demonstrate that cycling cardiomyocytes preferentially occur in the left side of the ventricular septum rather than its right side, both in homeostasis and after ischemic injury. Considering that blood pressure is known to be higher in the left ventricle than the right ventricle^26^, we examined whether altering chamber pressure can induce cardiomyocyte cell-cycle activity in the adult heart. Pursuing this, we exposed the ProTracer mice to pressure overload using the transverse aortic constriction (TAC) model (Fig. 8a)^27^. Tamoxifen was administered on adult mice of 8-10 weeks old (set as 0w), and TAC was performed after 1 week, and hearts were collected for analysis at 7 weeks after TAC (Fig. 8a). Confirming successful model establishment, the cardiomyocyte size was significantly increased after TAC as compared with the sham operation group (Fig. 8b). Our finding that there were significantly more GFP^+^ cardiomyocytes in the TAC group than in the sham group (~5 times, Fig. 8c, d) indicated that increased pressure can promote cardiomyocyte cell-cycle activity. We also noticed a highly enriched GFP^+^ CMs in the upper region of the ventricular septum after TAC (Fig. 8c), indicating that increased cell cycle activity (potentially some new cardiomyocytes) in addition to cell hypertrophy may contribute to the thickening of the ventricular septum, an abnormality usually observed in hypertrophic obstructive cardiomyopathy^28^. Thus, it is probable that cardiomyocytes in the subendocardial regions of the LV under normal condition may sense the relatively higher pressure experienced at this region, after which they can appropriately respond by initiating cellular signaling program(s) that ultimately activate their cell cycle activity. We also tried to find the diversity of cycling activities when primed ProTracer at young or old age (Supplementary Fig. 8a, b). It turned out that there were no significant difference in the number of cycling cardiomyocytes between 20 W and 64 W mice (Supplementary Fig. 8c, d). Previous study using ^14^C incorporation indicated that in human the yearly rate of cardiomyocyte renewal declined from 1% at the age of 25 to 0.45% at the age of 75^4^. Difference between our results and Bergmann’s may have some factors. Firstly, there could be difference between ^14^C carbon dating and lineage tracing methods. Our genetic system records cycling cardiomyocytes which include both divided and non-divided cardiomyocytes. Due to this limitation, ProTracer does not directly measure cardiomyocyte division. Secondly, different study models (human and mouse) may have variation in species. Immunostaining for GFP and TNNI3 on whole-heart sections revealed that regional cycling cardiomyocyte pattern still maintained even the ProTracer were primed at old age (Supplementary Fig. 8e–g).

### Hippo pathway effector YAP regulates regional cardiomyocyte cell-cycle activity

The Hippo pathway is known to mediate many aspects of organogenesis and organ homeostasis—including cardiomyocyte proliferation^11,29–31^. For molecular regulation of cell proliferation, Hippo signaling pathway has recently been reported to inhibit cell proliferation through suppressing the activity of transcriptional effector YAP through phosphorylation^29,32^. Here, we first treated adult *Ki67-CrexER;R26-GFP* mice of 8 weeks old with AAV9-Dre (Fig. 8e). We then used Yap-shRNA to knockdown YAP in cardiomyocytes by AAV9 at 1 week after Dre virus treatment, and collected hearts 4 weeks later (Fig. 8e). Compared with AAV9-Control, AAV9-shRNA treated hearts had profound reduction in GFP^+^ cardiomyocytes in the subendocardial regions, and there was no noticeable regional cardiomyocyte cell-cycling patterning formation (Fig. 8f). The above data indicated that Hippo effector YAP regulates cell-cycling of cardiomyocytes in the adult heart.

## Discussion

Cardiomyocyte renewal for repairing the injured heart has gained more attention these years, and the field has reached consensus on cardiomyocyte proliferation instead of stem cell as sources for new cardiomyocytes^33,34^. Developing methods for tracing in vivo cardiomyocyte proliferation is fundamental for exploring the mechanisms of heart repair and regeneration, and also provides new means of exploring therapeutic approach to promoting cardiomyocyte proliferation. In the present study we developed a genetic system to specifically monitor and record cycling cardiomyocytes in situ, and identified an unexpected trend: cycling cardiomyocytes in adult mouse hearts are highly regionalized. The exact reason behind this trend is not clear, but several hypotheses are plausible. The enrichment of cycling cardiomyocytes in the papillary muscle might be associated with the relatively stronger mechanical force experienced in this region (i.e., compared to the papillary muscles in the RV attached to the tricuspid valve), which must withstand the full force of the LV contraction during ventricular systole^35^. While we did show that increasing the pressure does significantly promote increase of cycling cardiomyocytes, it remains unclear if this results from a direct or indirect regulatory mechanism; further experiments which monitor both the post-mechanical-stress viability and functions of the relevant cell types in this heart region could help clarify the specific impacts of mechanical force on cell cycle activity and proliferation. Additionally, reason of the higher preference for cycling cardiomyocytes in the subendocardial region is not known. During normal development, there is a bust of cardiomyocyte proliferation in the subendocardial region^36,37^. Our observation in adult hearts may mirror that of normal heart development albeit at a significantly lower rate. It is interesting to know if these regional pattern could be due to the special origin of cardiomyocytes during development. However, our current ProTracer system is not able to discern whether cycling cardiomyocytes are derived from any specific cardiac progenitors located during cardiac formation. The subendocardial location of cycling cardiomyocyte may indicate their origin of a subset of cardiac progenitors derived from the second heart field^38^ or cardiac neural crest cells (CNCs). Recent studies reported that CNCs generate a number of trabecular cardiomyocytes that undergo multiple clonal division during compaction, and also continue to generate cardiomyocytes after birth, suggesting that postnatal heart sustains cardiomyocyte-producing CNCs^39^. Others have suggested that differential oxygen levels in the sub-endocardium vs. the outer ventricular layer may enhance cardiomyocyte cell cycle activity^40^. Nonetheless, our observations provide the basis for future mechanistic studies to determine the triggering events that account for certain cardiomyocytes to undergo cell-cycle activity and also, for some of them, cell division in distinct locations.

As mature cardiomyocytes in adult mammalian hearts could be polyploidy^41^, the caveat and limitation of using ProTracer system for cardiomyocyte study is that the expression of cell cycle markers Ki67 or Ccna2 does not necessarily denote complete cytokinesis or cell division of cardiomyocytes, albeit they have been widely used for study cell proliferation. Considering that any cardiomyocyte undergoing cytokinesis should have expressed cell cycle genes such as *Ki67*, genetic fate mapping of Ki67^+^ cells captures those divided cardiomyocytes as well as other non-dividing but cycling cardiomyocytes with polyploidy. While it is technically challenging to exclusively trace cardiomyocyte division at current stage, *Ki67*- or *Ccna2*-ProTracer does help identify cycling cardiomyocyte populations, which include, and also are enriched for, cytokinesis events from whole cardiomyocyte population. Our clonal analysis of Ki67^+^ cardiomyocytes labeled sparsely by ProTracer revealed that a subset of labeled cardiomyocytes can undergo complete cell cycle by generating new cardiomyocytes. Indeed, the high signal-to-background resolution for in situ GFP^+^ cardiomyocyte detection by ProTracer in clonal analysis (sparse labeling experiment) revealed some isolated pairs of cardiomyocytes in heart tissues, where two neighboring GFP^+^ cardiomyocytes are side-by-side among all other GFP^–^ cardiomyocytes (Fig. 3). Given the known rarity of GFP^+^ cardiomyocytes (cell-cycle positive) by sparse labeling strategy, the occurrence of two neighboring GFP^+^ cardiomyocytes strongly indicates two post-cytokinesis daughter cells. Our results also revealed that, of GFP^+^ cardiomyocytes, ~ 13% are detected in the 2-cell clusters and also here revealed regional pattern for these GFP^+^ divided cardiomyocytes in the adult heart. These results provide direct genetic evidence that cardiomyocytes in adult hearts can undergo full cell cycle for generation of new cardiomyocytes during homeostasis and after injuries. In future, this ProTracer system can be iterated and improved to more specifically illuminate those divided cardiomyocytes throughout the entire heart, e.g. by incorporating mosaic analysis with double markers^42^, rainbow systems^43,44^, or potential technologies that could distinguish two neighboring cells.

## Methods

### Mice

All mice were used in accordance with the guidelines of the Institutional Animal Care and Use Committee of Shanghai Institute of Biochemistry and Cell Biology, Chinese Academy of Sciences. The *R26-DreER, R26-GFP, Tnnt2-DreER, Tnnt2-Dre, R26-rox-tdTomato, ACTB-Cre*, *R26-RSR-LSL-tdTomato* mouse lines have been reported previously^19–21,45–47^. The *Ki67-CrexER* knock-in mouse line was generated by knocking the cDNA sequence encoding CrexER, which contains the Cre recombinase and a rox-flanked mutant form of the estrogen-receptor hormone-binding domain, into the 3' UTR of Ki67 gene. We removed the translational stop codon of Ki67 gene and used the self-cleaved peptide P2A to link the CrexER cDNA. For the *Ccna2-CrexER* mouse line, the cDNA sequence encoding CrexER was inserted at the site encoding the translational stop codon of *Ccna2* gene using the self-cleaved peptide P2A. *Tnnt2-LSL-mTnG* mouse line was generated by knocking the loxP-Stop-loxP-membrane tdTomato-nuclear GFP cDNA into the 5' UTR of *Tnnt2* gene. The *Ki67-CrexER*, *Ccna2-CrexER*, and *Tnnt2-LSL-mTnG* mouse lines were generated by Shanghai Model Organisms Center, Inc. (SMOC). All mice were maintained on a 129, C57BL6, and ICR mixed background. These mice at age of 8–10 weeks old were treated with 0.1–0.2 mg/g tamoxifen by gavage (Sigma, T5648, 20 mg/ml dissolved in corn oil), AAV9-Dre virus intravenously or EdU incorporation by drinking water. Mice of both male and female were randomly assigned to different experimental groups in all experiments. And mice were not excluded during all these experiments.

### Genomic PCR

Genomic DNA was prepared from the embryonic yolk sac or the transgenic mouse tails. Tissues were lysed by incubation with lysis buffer (100 mM Tris–HCl, PH 7.8, 5 mM EDTA, 0.2% SDS, 200 mM NaCl and 100 µg/ml proteinase K) at 55 °C overnight, followed by centrifugation at maximum speed for 8 min to obtain supernatant with genomic DNA. Genomic DNA was precipitated with isopropanol, washed in 70% ethanol, and dissolved in deionized water. All the embryos and mice were genotyped using genomic PCR as described previously^19–21,45–47^. The genomic PCR primer sequences were provided in the supplementary table 1.

### AAV treatment

cDNA sequence encoding Dre recombinase followed by a WPRE cassette was cloned into the AAV9 plasmid, which was driven by the cardiac TNT promoter, to yield AAV9-Dre over-expression vector^22^. AAV9-Dre virus was generated by transferring over-expression vector and auxiliary plasmids to AAV-293 cells. The AAV9-Dre virus was produced and purified using a standard protocol with modifications. A scrambled DNA sequence was used cloned into AAV9 plasmid as AAV9-control. Genomeditech (Shanghai, China), Taitool Bioscience (Shanghai, China), and OBIO Technology (Shanghai, China) provided support in producing AAV9-Dre and AAV9-control virus. 2 ~ 4 × 10^11^ titer of virus was intravenously injected into mice. For sparse labeling, about 5 × 10^10^ titer of virus was intravenously injected into mice. For in vivo knocking down YAP, two designed Yap-shRNA sequences were cloned into GPAAV-HU6-MCS-WPRE plasmid with U6 promoter and H1 promoter respectively. AAV9-shYAP virus was generated by co-transferring the constructed plasmids and auxiliary plasmids into AAV-293 cells, followed by purifying with a standard protocol with modifications. About 5 × 10^11^ titer of AAV9-shYAP or AAV9-control virus was intravenously injected into mice. Genomeditech (Shanghai, China) provided support in producing AAV9-shYAP and AAV9-control virus. The two designed Yap-shRNA sequences were GATCCGCTGATGAATTCTGCCTCAGGCTCGAGCCTGAGGCAGAATTCATCAGCTTTTTT and GATCCGGAGAGACTGCGGTTGAAACACTCGAGTGTTTCAACCGCAGTCTCTCCTTTTTT.

### Whole-mount fluorescence microscopy

Mouse hearts or embryos were washed in phosphate-buffered saline (PBS) and fixed in 4% paraformaldehyde (PFA) at 4 °C for 20 min or 1 h (depending on tissue size) followed by three times of PBS wash. The tissues were placed on agar to obtain whole-mount bright-field and fluorescence images by Zeiss stereoscope (Axio Zoom.V16).

### Immunostaining

Immunostaining was performed according to protocols described previously^48^. Embryos or hearts were collected in ice-cold PBS and then fixed with 4% PFA at 4 °C for 20 min to 1 h depending on the tissue size. After three times washing with PBS, tissues were dehydrated with 30% sucrose dissolved in PBS at 4 °C overnight. Then the tissues were embedded in optimum cutting tissue (O.C.T., Sakura) and stored at –80 °C before sectioning. Cryosections (~ 10μm) were collected on negatively charged slides and stored at –20 °C before use. The dried sections were washed twice for 15 min with PBS and then blocked with 5% normal donkey serum in PBST (0.2% Triton X-100 in PBS) at room temperature for 30 min. Sections were incubated with primary antibodies at 4 °C overnight and then washed with PBS three times followed by incubation with fluorescence conjugated secondary antibodies (Invitrogen or Jackson ImmunoResearch) at room temperature for 30 min. Sections were then washed with PBS three times and mounted with a mounting medium containing DAPI (Vector Labs). For staining of dissociated cells, the dissociated cardiomyocytes were first fixed with 4% PFA at 4 °C for 15 min, then collected using 200 g centrifugation for 3 min, with the following steps being the same as section immunostaining. For weak signals, we used horseradish peroxidase- or biotin-conjugated secondary antibodies and detected the signals by using a tyramide signal amplification kit (PerkinElmer). Primary antibodies used in immunofluorescence for this study included: ACTN2 (Sigma, A7811-.2 ML, 1:500), GFP (Invitrogen, A11122, 1:500), TNNI3 (Abcam, AB56357, 1:200), WGA (Invitrogen, W32466, 1:1000), ESR (Abcam, Ab27595, 1:1), tdTomato (Rockland, 600-401-379, 1:1000), PECAM (BD Pharmingen, 553370, 1:500), PDGFRa (eBioscience, 14-1401-81, 1:500), E-Cad (R&D, AF748, 1:500), YAP1 (ABclonal, A1002, 1:500), Ki67 (Thermo scientific, RM-9106-S0, 1:100), Nkx2.5 (R&D, AF2444, 1:100). The included secondary antibodies were Alexa donkey anti-mouse 555 (Invitrogen, A31570, 1:1000), Alexa donkey anti-rabbit 488 (Invitrogen, A21206, 1:1000), Alexa donkey anti-goat 555 (Invitrogen, A21432, 1:1000), Alexa donkey anti-goat 647 (Invitrogen, A21447, 1:1000), Immpress anti-rabbit immunoglobulin (Vector lab, MP-7401-50, 1:1), TSA Cyanine 3 System (PerkinElmer, NEL744A001KT, 1:1000), Alexa donkey anti-rabbit 555 (Invitrogen, A31572, 1:1000), Alexa donkey anti-rat 555 (Invitrogen, A21434, 1:1000). Nuclei dyes included: DAPI (D9542-1MG, 1:1000), Propidium Iodide (P4170-10MG, 1:1000). Images were taken with Nikon A1 confocal system or a Zeiss stereo microscope (Axio Zoom.V16). The obtained images were analyzed by ImageJ (NIH) software. The images were merged with the Image color-merge channels function, and the sticks were performed using Z-projects and max intensity projection. In the stack, *XZ* and *YZ* axes signals were shown by orthogonal view. Merged signals and split channels were used to delineate the signals at single-cell resolution as described previously. The consecutive Z-stack confocal images were obtained from Olympus FVMPE-RS and analyzed with ImageJ (NIH) software.

### EdU Staining

Intraperitoneally injected 10–50 µg/g EdU (ThermoFisher, A10044) into mice, 3 h before mouse sacrifice. In the long-term incorporation system, EdU was dissolved in drinking water at 0.3 mg/ml. Tissue sections were blocked in PBSST (5% normal donkey serum in PBS containing 0.2% Triton X-100) for 30 min at room temperature. The immunofluorescence staining was performed with the Click-iT cocktail (Invitrogen, C10340) according to the manufacturer’s instruction.

### Cardiomyocyte quantification

The whole hearts were sectioned and mounted on 50–60 negatively charged slides, each containing 8–10 heart sections. About 40–50 heart tissue sections representatively of each heart were stained with GFP, TNNI3, and WGA. The percentage of GFP^+^ cardiomyocytes was quantified as the number of GFP^+^TNNI3^+^ cardiomyocytes divided by TNNI3^+^ cardiomyocytes. A researcher who was blinded to the project counted the number of GFP^+^ cardiomyocytes by Olympus fluorescence microscope (BX53). 5 hearts were quantified for each group.

### Cardiomyocyte isolation

Cardiomyocytes were isolated as described previously^47^. Briefly, mice were injected with 200 μl heparin (6.25U/μl) intraperitoneally to prevent blood coagulation. Thirty minutes later, sodium pentobarbital (80 mg per kg body weight) was injected intraperitoneally to anesthetize mice. Then dissected hearts were perfused with perfusion buffer (modified Tyrode’s solution (MTS), 137 mM NaCl, 4 mM KCl, 0.33 mM NaH_2_PO_4_, 1 mM MgCl_2_, 10 mM HEPES, 5 mM taurine, 10 mM 2,3-butanedione monoxime (BDM, B0753, Sigma) and 10 mM glucose, PH = 7.4) at a flow rate of 4 ml/min for about 5 min through the aorta. Then the hearts were perfused with digestion buffer (MTS containing 250 U/ml collagenase type 2) (Worthington, LS004176) and 3 U/ml Protease XIV (Sigma, P5147) for about 15 min at the same flow rate. The digested hearts were transferred to transfer buffer (MTS containing 0.5 mg/ml BSA) and minced with forceps followed by filtering through a 100 μm strainer. Isolated cells were centrifuged with 20 g for 3 min at 4 °C to collect cardiomyocytes. The cell pellets were re-suspended with 50% (vol/vol) Percoll (Sigma, P1644) in transfer buffer and centrifuged with 100 g for 5 min at 4 °C to further separated the dead cardiomyocytes, non-myocytes, and live cardiomyocytes. The supernatant contains the dead cardiomyocytes, non-myocytes, and cell debris, discard them. Re-suspended the pellets which contain the live cardiomyocytes with transfer buffer for further analysis.

### Southern blotting

Genomic DNA was prepared from dissociated cardiomyocytes of the indicated mice. Genomic DNA from *Ki67-CrexER* cardiomyocytes was digested into 4.6 kb fragments by restriction enzyme ApaLI (NEB), while DNA from *Ki67-Crex* cardiomyocytes was digested into 3.6 kb fragments. The probe was designed based on the Cre region of the CrexER DNA sequence and the sequence was provided in the supplementary table 1. Shanghai Model Organisms Center, Inc. (SMOC) provided support in the Southern blotting experiments.

### Flow cytometry

Dissociated cardiomyocytes were centrifuged and incubated with red-blood-cell lysis buffer (2–5 ml, eBioscience, 00-4333-57) for 5 min at room temperature. After washing with an equal volume of isolation buffer (2 mM EDTA and 0.25% BSA in PBS), cells were centrifuged at 20 g for 3 min. The re-suspended cells were first stained with LIVE/DEAD Fixable Violet Dead Cell Stain Kit (Life Technology, L34955) according to the manufacturer’s instruction. Then cells were washed with isolation buffer and centrifuge with 20 g for 3 min, and the pellets were blocked in Fc block (eBioscience, 14-0161, 1:100) for 5 min at room temperature and then directly incubated with antibodies mixture containing CD31 APC (eBioscience, 17-0311-80, 1:40), CD140a APC (eBioscience, 17-1401-81, 1:500), CD45 APC (eBioscience, 17-0451-82, 1:400) at 4 °C for 30 min. After washing with isolation buffer, cells were re-suspended with 500 µl isolation buffer and finally analyzed with FACS Aria II Flow Cytometer (BD Biosciences). The raw data were processed by FlowJo software (Tree star).

### Cardiac injury model

Myocardial infarction (MI) was performed on adult mice as described previously^49^. Mice for sham or MI surgery were randomly allocated. Mice were anesthetized with isoflurane (2%) mixed with 100% O_2_ (at the flow rate at 0.5–1 L/min) and put on a heated pad. When they do not respond to a tail and toe pinch, fixed the limbs on the pad and disinfected the chest followed by tracheal intubation. A vertical 1-cm incision was made between the third and fourth intercostal ribs to expose the internal chest. To avoid the injury of blood vessels, muscle and fascia were separated by blunt dissection. Then the left anterior descending branch of the coronary artery was ligated with a 0-8 suture. When the heart appeared the signs of cyanosis, the ligation was successful. Then closed the incision and discharged the air from the chest. The incision was disinfected and mice were supplied with pure oxygen for 4-5 min until they resuming spontaneous breath, then removed the tracheal intubation. Mice were kept warm until they resumed normal behavior. In the sham group, the same operations were performed except for the ligation of LAD. The investigator who performed the surgery was blinded to the allocation.

Transverse aortic constriction (TAC) was performed on adult mice as previously described^27^. Adult mice were randomly allocated to the sham or TAC group. A researcher who was blinded to the mouse genotype and treatment performed the surgery. Isoflurane (2%) mixed with 100% O_2_ (at the flow rate at 0.5–1 L/min) was used to anesthetize mice. In all 125–150 breaths per minute and a tidal volume of 0.1–0.3 ml was promised to keep mice alive. The isoflurane concentration was adjusted to 1.5% upon reaching a surgical plane of anesthesia. After chest skin disinfection and series intubation, thymus, and fat tissues were separated to expose the aortic arch. After placing a 6.0 silk suture between innominate and left carotid artery and tying two loose knots around the transverse aorta, a small piece of a 27^1^/_2_ gauge blunt needle was placed parallel to the transverse aorta. To yield a constriction at 0.4 mm in diameter, the suture was quickly tied against the needle and the needle was removed promptly. After the TAC surgery, mouse skin was closed using a 6.0 suture. The closed mouse skin was disinfected again and mice were kept warm after they resuming spontaneous breath. The sham group was performed in the same manner but the suture was not tied against the needle. Seven weeks after injury, mouse hearts were collected for further studies.

### Sirius red staining

The cryo-sections were dried and washed with PBS twice. The following procedures were performed at room temperature. Then the sections were fixed with Bouins’ solution overnight. The sections were washed with tap water until the yellow color disappeared. Then sections were fixed with Fast green for 3 min and then washed with tap water, followed by fixed with 1% Acetic acid for 1 min. After washing with double distilled water, the sections were fixed with 0.1% Sirius red for 1 to 1.5 min. Then the sections were washed with double distilled water and dehydrated. Dehydrate procedure included: Fixed in 95% ethanol for 3 min twice, in 100% ethanol for 3 min twice, and in xylene for 5 min twice. The sections were covered by Permount^TM^ Mounting Medium.

### Cardiomyocyte nucleation analysis

The resuspended live cardiomyocytes were stained with Hoechst 33342 (Beyotime biotechnology, C1022, 10ug/ml) at 37 °C for 15 min. After washing with transfer buffer, cardiomyocytes were resuspended with transfer buffer. Nuclear and GFP fluorescence signals were detected by Olympus BX53. The karyotype determination of a nucleus (diploid or polyploid) was based on the size of the nucleus.

### Statistical analysis

All data were obtained from four to five independent experiments, as mentioned in each figure legend. Data are presented as mean values ± s.e.m. Statistical analysis for data from two groups was performed using an unpaired Student’s *t*-tests. *P* < 0.05 was considered as statistically significant.

### Reporting Summary

Further information on research design is available in the Nature Research Reporting Summary linked to this article.

## Supplementary information

Supplementary Information

Reporting Summary

## Data Availability

All data generated by this study are included in this article and its supplementary materials. They are available upon request. Source data are provided with this paper.

## Source data

Source Data
