# Supplementary material for: Cell proliferation fate mapping reveals regional cardiomyocyte cell-cycle activity in subendocardial muscle of left ventricle
Source: Nat Commun. 2021 Oct 1;12:5784. doi: 10.1038/s41467-021-25933-5 (PMC8486850; doi:10.1038/s41467-021-25933-5)
Supplement: Supplementary file 1 — Supplementary Information [file 41467_2021_25933_MOESM1_ESM.pdf]

Supplementary Material for

Cell proliferation fate mapping reveals regional cardiomyocyte cell-cycle activity in subendocardial muscle of left ventricle

Xiuxiu Liu, Wenjuan Pu, Lingjuan He, Yan Li, Huan Zhao, Yi Li, Kuo Liu, Xiuzhen Huang, Wendong Weng, Qing-Dong Wang, Linghong Shen, Reza Ardehali, Ben He, Bin Zhou

Supplementary Figure 1 to 8

Supplementary table 1

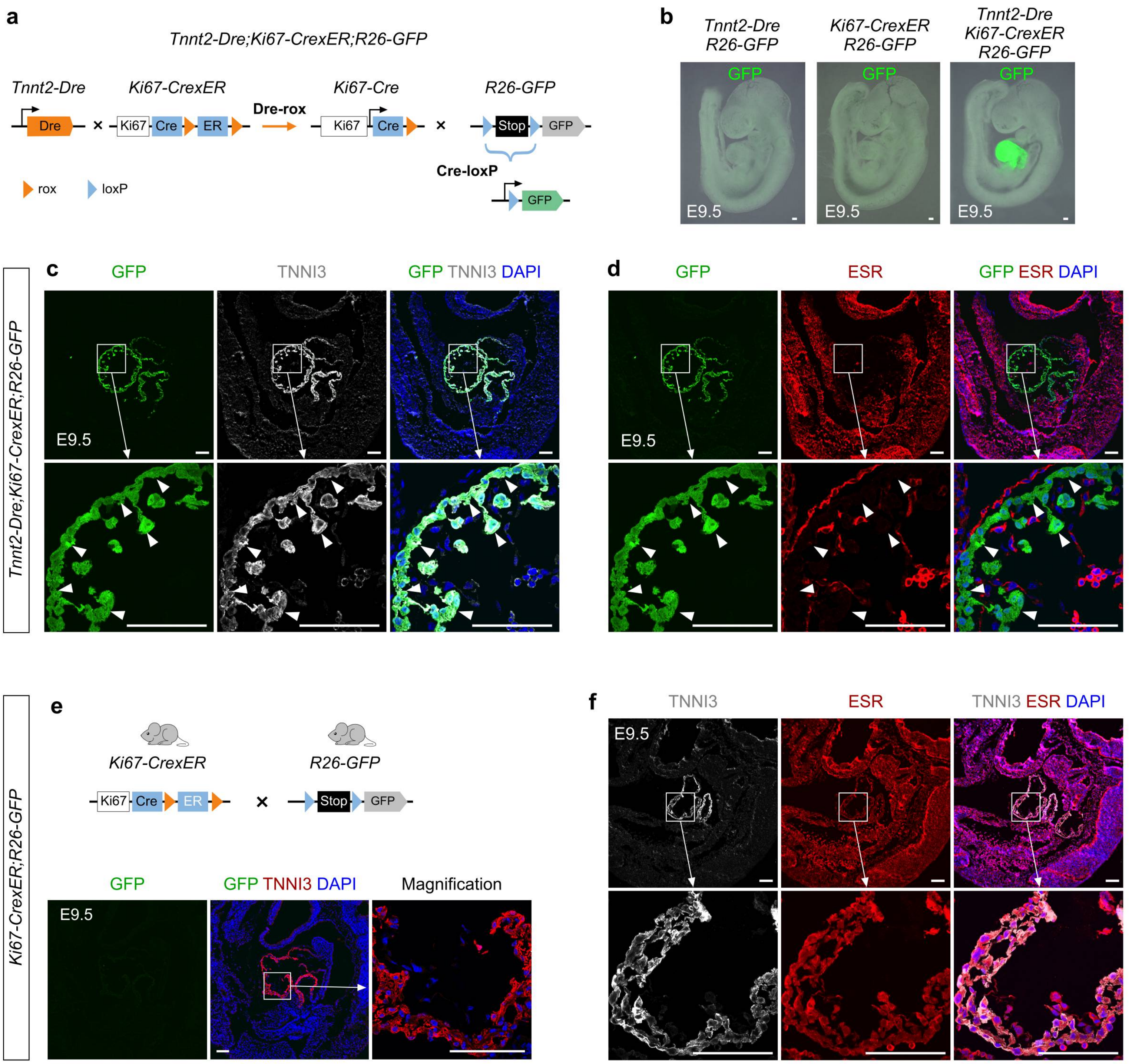

Supplementary Fig. 1. Characterization of *Ki67-CrexER* line by Dre-rox recombination.

a Schematic showing strategy for *Tnnt2-Dre* primed ProTracer system. **b** Whole-mount fluorescence images of E9.5 *Tnnt2-Dre;R26-GFP*, *Ki67-CrexER;R26-GFP*, or *Tnnt2-Dre;Ki67-CrexER;R26-GFP* embryos. **c**, **d** Immunostaining for GFP and TNNI3 (**c**) or ESR (**d**) on same section of E9.5 *Tnnt2-Dre;Ki67-CrexER;R26-GFP* embryo shows ER is not detectable after its excision in GFP^{+}TNNI3^{+} cardiomyocytes (arrowheads). **e**, **f** Immunostaining for TNNI3 and GFP (**e**) or ESR (**f**) on E9.5 *Ki67-CrexER;R26-GFP* embryonic sections shows that, while there is no *Tnnt2-Dre* allele, ER is maintained in cardiomyocytes. Scale bars: 100 μm. Each image is representative of 5 individual biological samples.

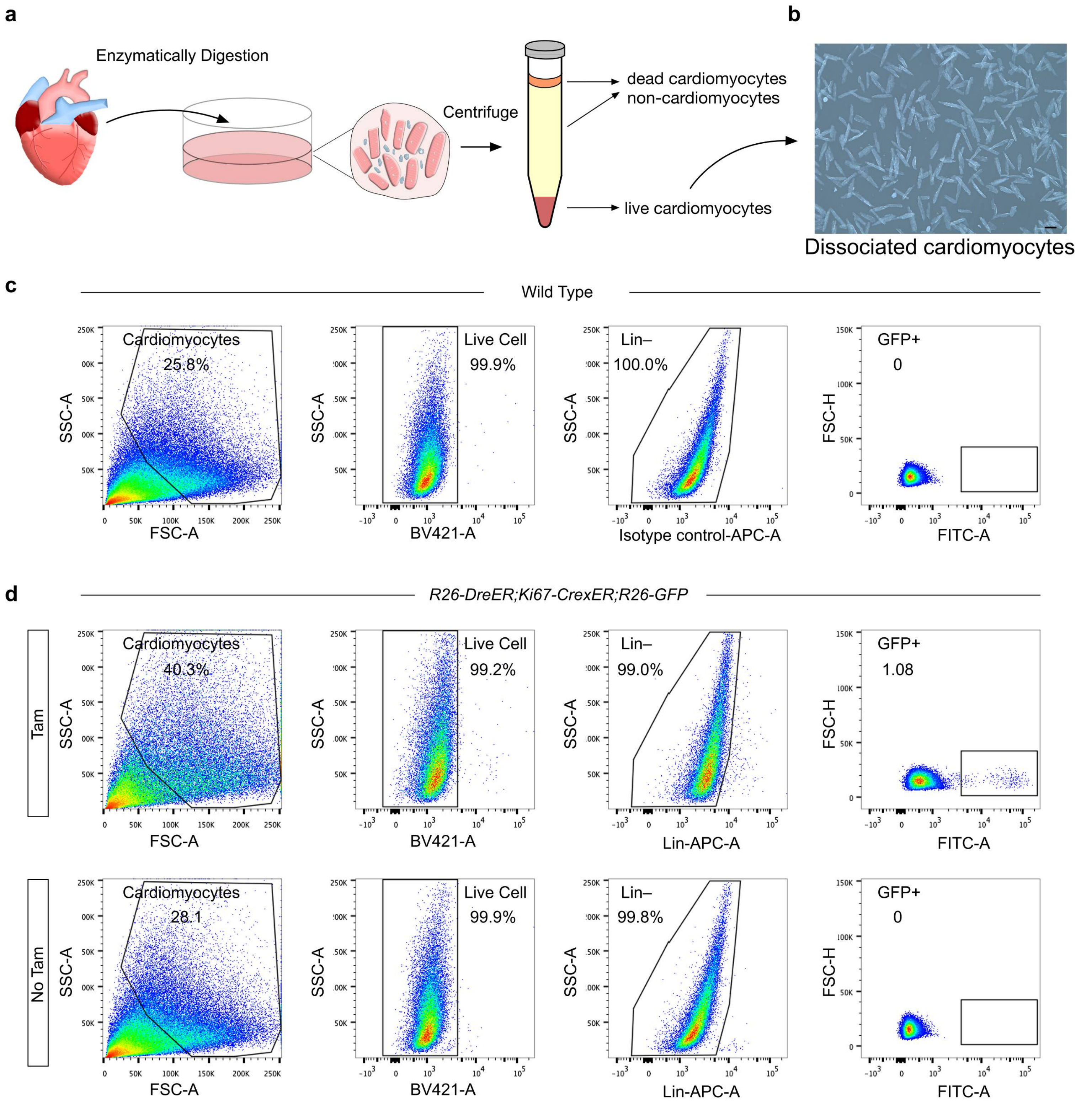

Supplementary Fig. 2. FACS gates for GFP+ cardiomyocytes in *R26-DreER;Ki67-CrexER;R26-GFP* mice.
a Schematic figure showing cardiomyocytes were enzymatically dispersed from hearts followed by centrifugation (see methods). **b** Image showing dissociated cells used for FACS analysis were cardiomyocytes. scale bars, 100 μm. The image is representative of 5 individual biological samples. **c** Successive gating shows sequential selection of cardiomyocytes by forward scatter (FSC) and side scatter (SSC). LIVE/DEAD Fixable Violet Dead Cell staining (BV421-A) was used to identify live cells. Cardiomyocytes of wild type mice were stained with isotype control antibodies as a control for staining of lineage-specific antibodies. **d** Same gating strategy was applied to cardiomyocytes of *R26-DreER;Ki67-CrexER;R26-GFP* mice with or without Tam treatment. Cardiomyocytes were stained with lineage-specific (Lin) antibodies against endothelial cells (CD31-APC), fibroblasts (CD140a-APC) and hematopoietic cells (CD45-APC) to allow exclusion of these cells. Lineage negative cells were gated for subsequent analysis. The FITC-A panels shown here correspond to Fig. 1e FITC-A panels.

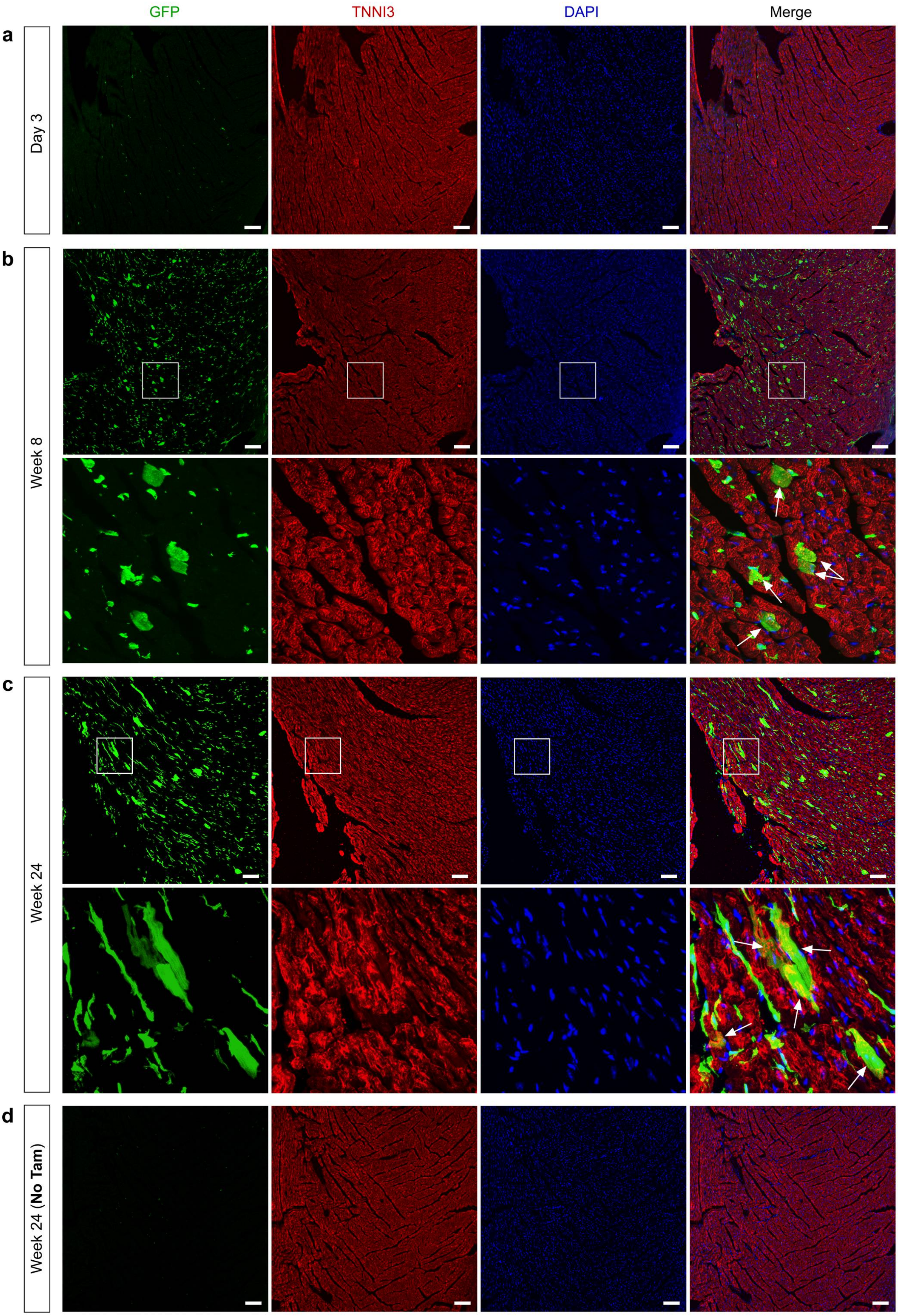

Supplementary Fig. 3. Ki67⁺ cells recorded by ProTracer in adult hearts.

a-**d** Immunostaining for GFP and TNNI3 on heart sections collected from *R26-DreER;Ki67-CrexER;R26-GFP* mice at Day 3 (**a**), Week 8 (**b**), and Week 24 (**c**) after tamoxifen treatment; or Week 24 after corn oil treatment (No Tam, **d**). Arrows, GFP^+ cardiomyocytes. Scale bars: 100 µm. Each image is representative of 5 individual biological samples.

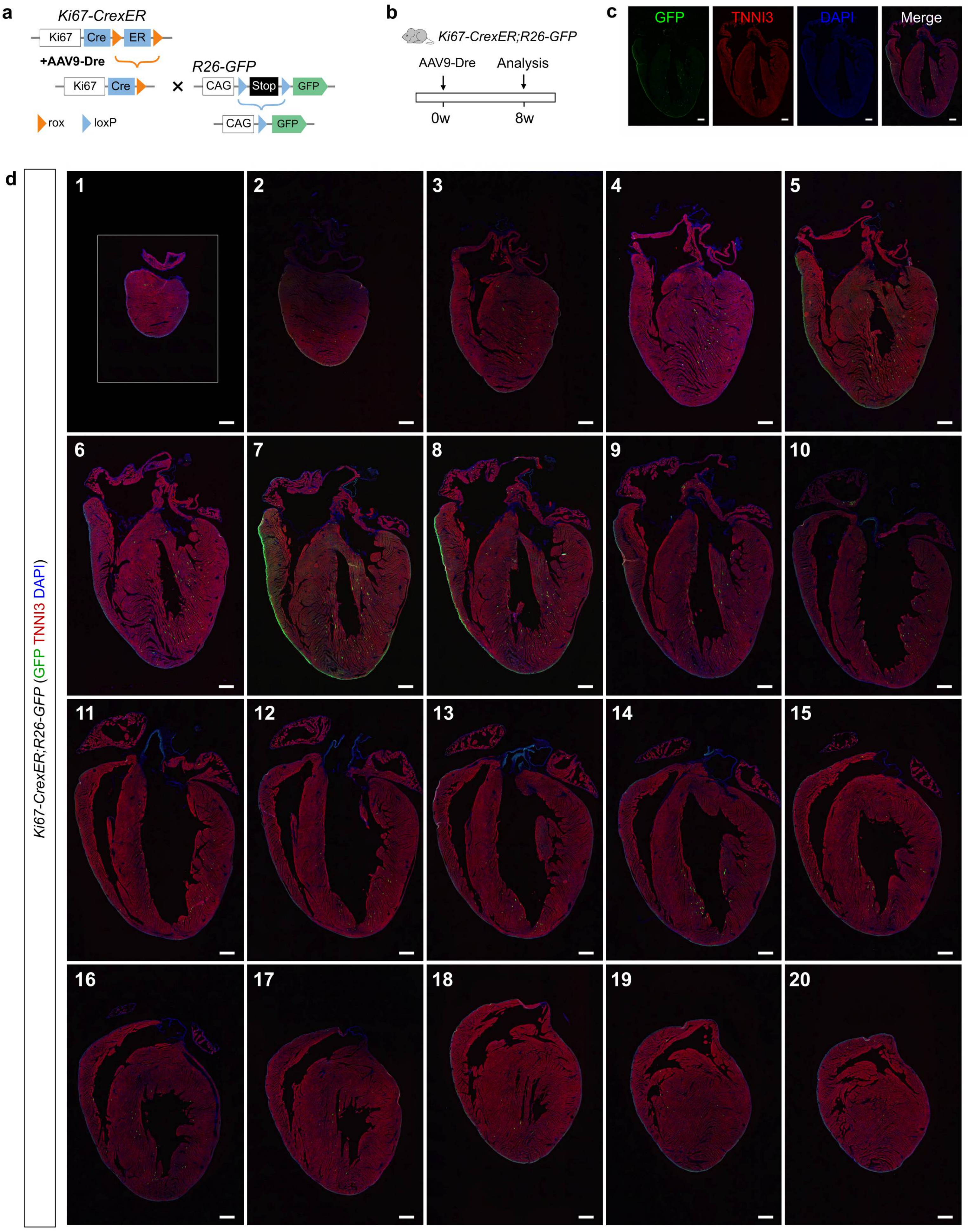

Supplementary Fig. 4. Cardiomyocyte cell-cycle activity recorded by *Ki67-CrexER;R26-GFP* hearts in series of sections.

a Schematic showing tracing strategy of cardiomyocyte-specific ProTracer. **b** Schematic showing experimental design. **c** Immunostaining for GFP and TNNI3 on heart sections with split channels. **d** Merged images of immunostained sections collected from *Ki67-CrexER;R26-GFP* hearts (1-20: from dorsal to ventral side). Scale bars: 500 μm. Each figure is representative of 5 individual biological samples.

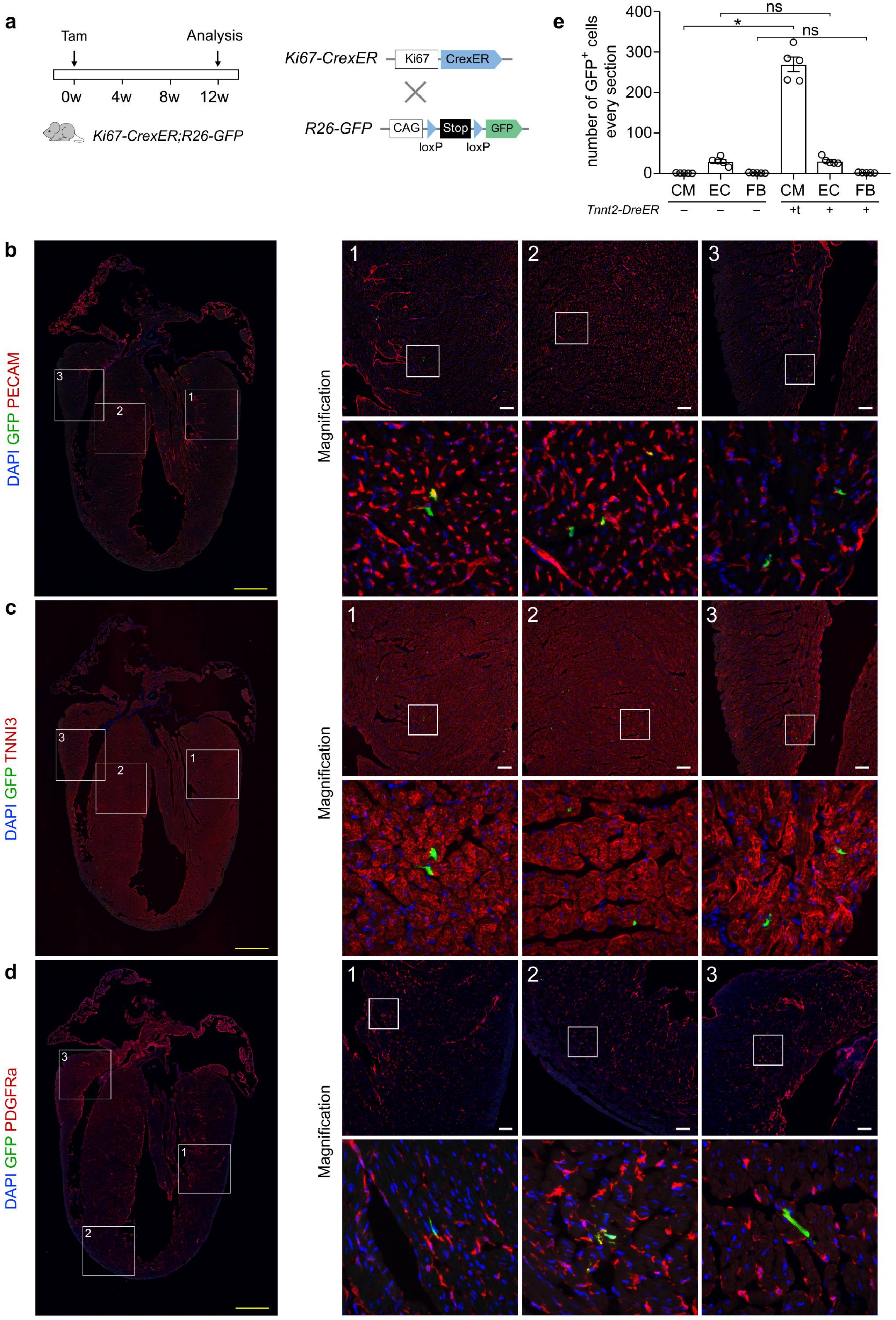

Supplementary Fig. 5. Examination of cell labeling by *Ki67-CrexER;R26-GFP* in adult hearts.
a Schematic figure showing experimental strategy using *Ki67-CrexER;R26-GFP* mice. **b**-**d** Immunostaining for GFP and PECAM (**b**), TNNI3 (**c**), and PDGFRa (**d**) on heart sections at Week 12 after tamoxifen treatment shows very few PECAM$^+$ endothelial cells or PDGFRa$^+$ fibroblasts expressing GFP, and almost no detectable GFP$^+$ cardiomyocytes. **e** Quantification of the number of different kinds of GFP$^+$ cells every heart section in *Ki67-CrexER;R26-GFP* or *Tnnt2-DreER*;*Ki67-CrexER;R26-GFP* mice. Data are the mean ± s.e.m.; n = 5. Data were analysed by two-tailed un-paired Student t-test, **P* < 0.0001, *P* = 0.8868, *P* = 0.2459 (CM, EC, FB); ns, non-significant. CM, cardiomyocytes; EC, endothelial cells; FB, fibroblasts. Scale bars, yellow, 1 mm; white, 100 µm. Each figure is representative of 5 individual biological samples.

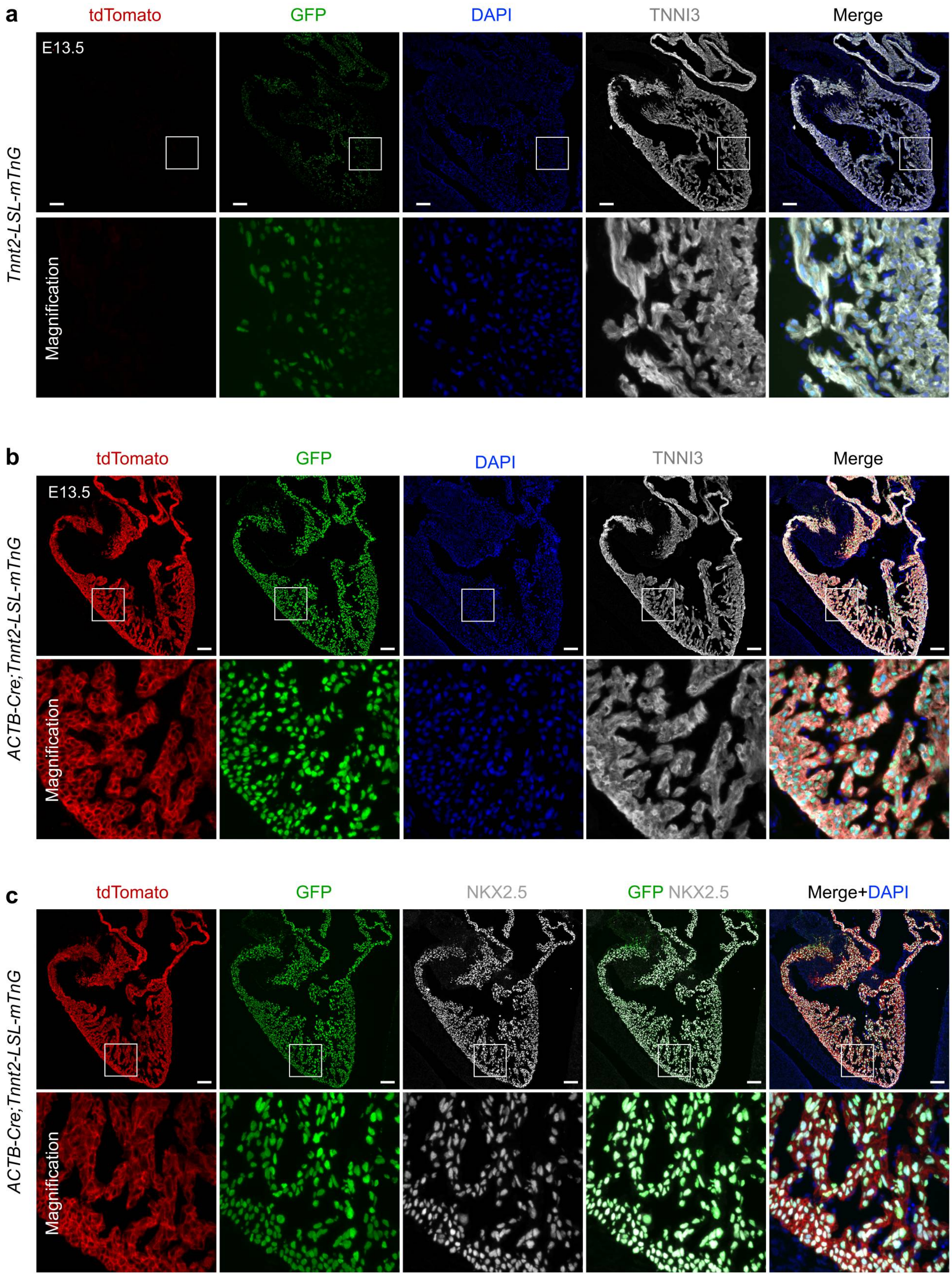

Supplementary Fig. 6. Generation and characterization of *Tnnt2-LSL-mTnG* line.
a Immunostaining for tdTomato, GFP, and TNNI3 on E13.5 *Tnnt2-LSL-mTnG* heart sections. Weak nGFP but not tdTomato signal is detected in TNNI3$^+$ CMs. **b**-**c** Immunostaining for tdTomato, GFP and TNNI3 (**b**) or NKX2.5 (**c**) on heart sections from E13.5 *ACTB-Cre;Tnnt2-LSL-mTnG* embryos. Robust nGFP and tdTomato signals are detected specifically in TNNI3$^+$ or NKX2.5$^+$ CMs. Scale bars, 100 μm. Each image is representative of 5 individual biological samples.

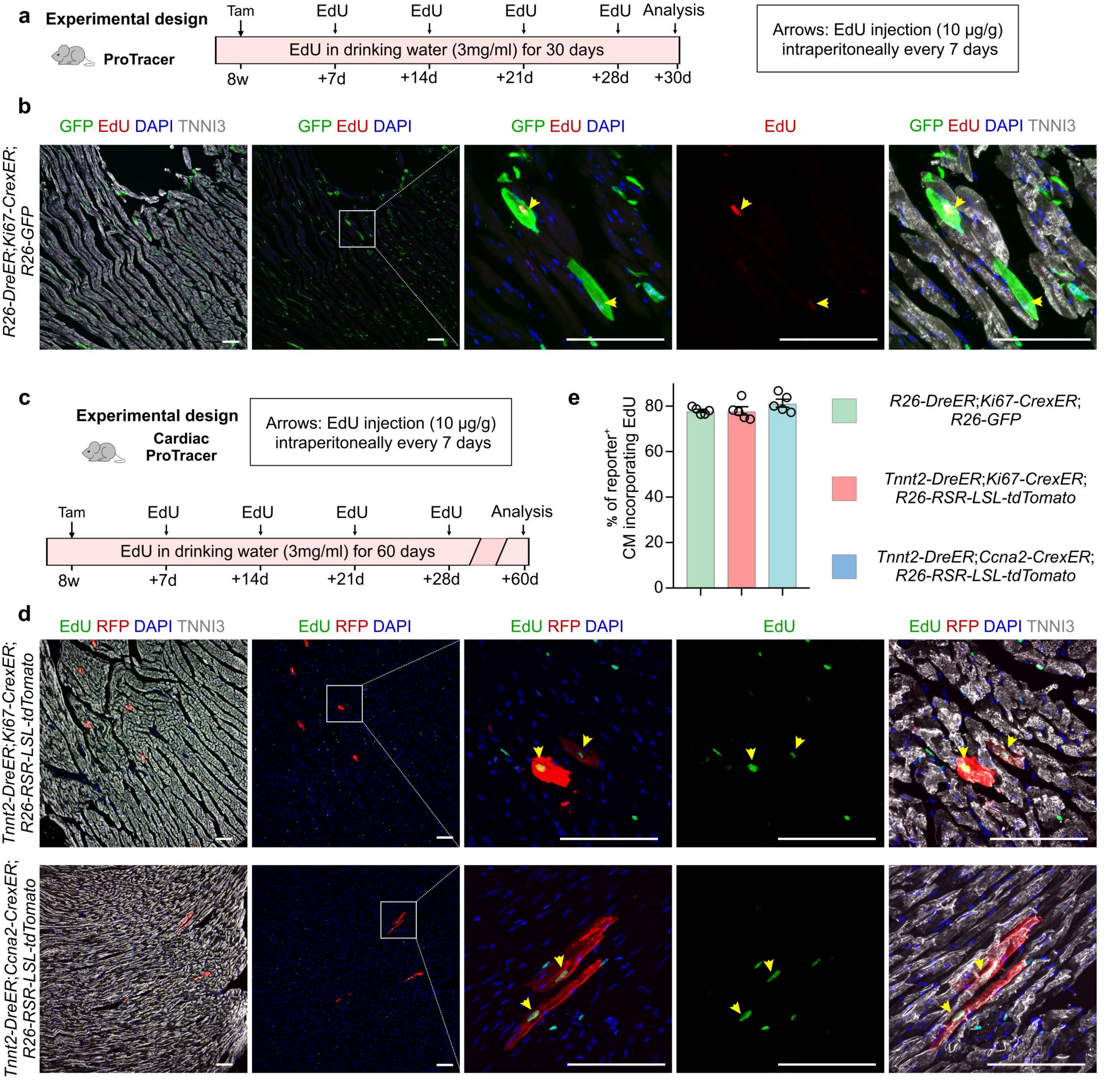

Supplementary Fig. 7. Examination of EdU incorporation in hearts of ProTracer mice

a Schematic figure showing experimental design of EdU incorporation on ProTracer mice. **b** Heart sections collected from EdU and tamoxifen treated *R26-DreER*;*Ki67-CrexER*;*R26-GFP* mice were stained with GFP, EdU, and TNNI3. Arrow heads, EdU^+GFP^+ cardiomyocytes. **c** Schematic showing strategy for EdU incorporation on cardiomyocyte specific ProTracer (Cardiac ProTracer). **d** Heart sections collected from EdU and tamoxifen treated *Tnnt2-DreER*;*Ki67-CrexER*;*R26-RSR-LSL-tdTomato* or *Tnnt2-DreER*;*Ccna2-CrexER*;*R26-RSR-LSL-tdTomato* mice were stained with EdU, RFP, and TNNI3. Arrow heads, EdU^+RFP^+ cardiomyocytes. **e** Quantification of percentage of $reporter^+$ cardiomyocytes incorporating EdU. Data are the mean ± s.e.m.; n = 5. Scale bars, 100 μm. Each image is representative of 5 individual biological samples.

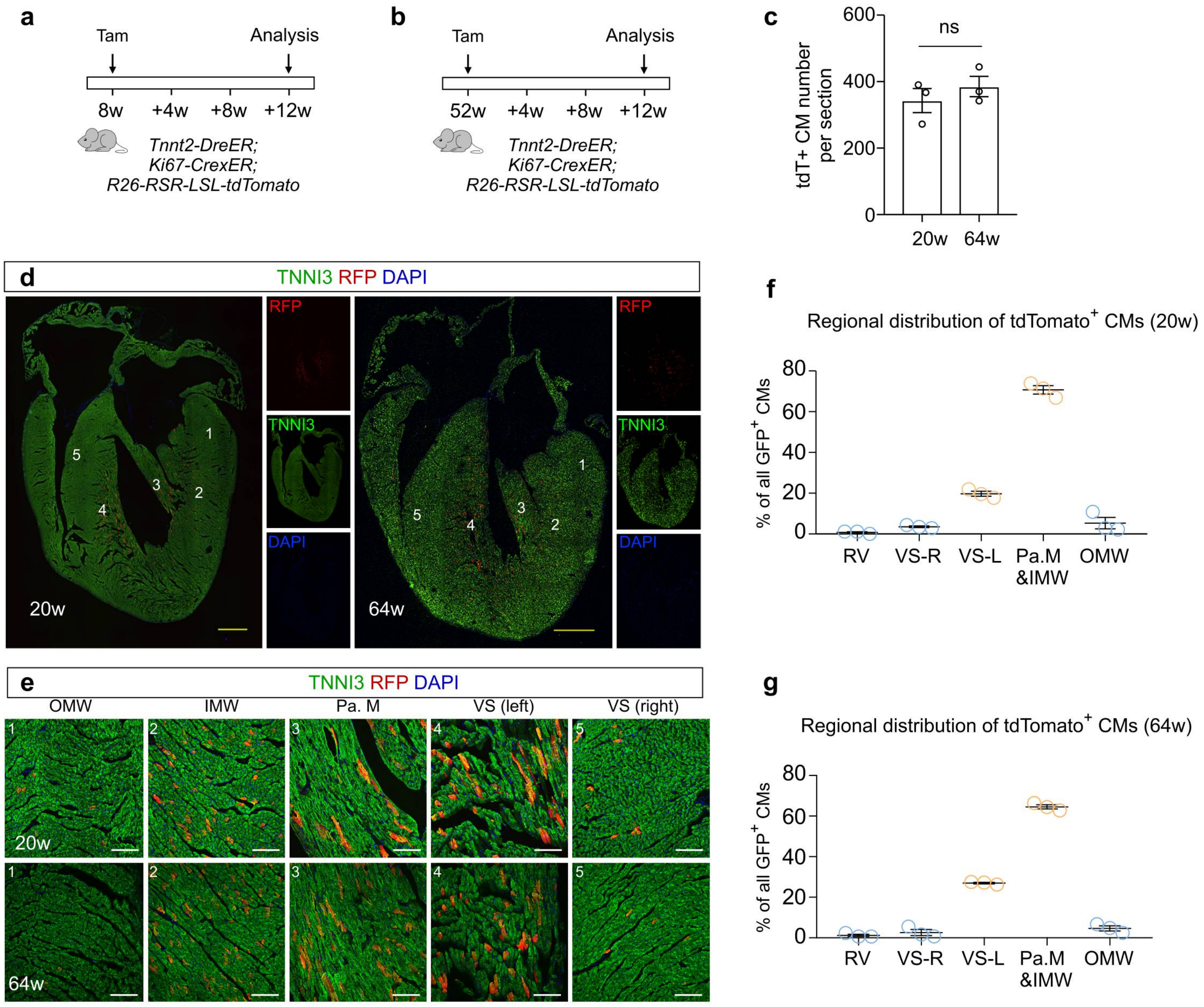

Supplementary Fig. 8. Cycling cardiomyocytes at old age

a-b Schematic figure showing experimental strategy using *Tnnt2-DreER;Ki67-CrexER;R26-RSR-LSL-tdTomato.* **c** Quantification of the number of tdTomato⁺ cardiomyocytes per heart section collected from *Tnnt2-DreER;Ki67-CrexER;R26-RSR-LSL-tdTomato* mice which were treated with tomoxifen at different time points. Data are the mean ± s.e.m.; n = 3. Data were analysed by two-tailed un-paired t-test. P = 0.42, ns, non-significant. **d** Immunostaining for TNNI3 and RFP on heart sections collected from *Tnnt2-DreER;Ki67-CrexER;R26-RSR-LSL-tdTomato* mice treated with tamoxifen at indicated time points. **e** Magnified views of heart sections in (d). **f-g** Quantification of the distribution of tdTomato⁺ CMs in different regions of the ventricles. Data are the mean ± s.e.m.; n = 3. Scale bars, yellow, 1mm; white, 100 µm. Each image is representative of 3 individual biological samples.

Supplementary table1: Genomic PCR primer list and Southern Blotting probe sequence

Genomic PCR primer list

Mouse line		
R26-DreER<br>M: 143bp<br>W: 364bp	M, forward	CGTGCTGGTTATTGTGCTGTCTC
	M, reverse	TACTCCTTGCCGATGTTCCTCAGG
	W, forward	TTGGAGGCAGGAAGCACTTG
	W, reverse	CCGACAAAACCGAAAATCTGTG
R26-GFP<br>M: 622bp<br>W: 297bp	M, forward	AAGGGAGCTGCAGTGGAGTA
	M, reverse	CCGAAAATCTGTGGGAAGTC
	W, forward	CAGCGACTTCTTCATCCAGAGC
	W, reverse	AAAGCAGCGTATCCACATAGCG
Tnnt2-DreER<br>M, 439bp<br>W, 773bp	forward	TCCGTGCCAGAATGAAAATGTC
	M, reverse	ACTCCTTGCCGATGTTCCTCAG
	W, reverse	TGTGGGGTGACTTGAGACCTAAGG
Tnnt2-Dre<br>M, 323bp<br>W, 422bp	forward	GCTGCCTTGCTGTGTTGTTCAG
	M, reverse	ACTCCTTGCCGATGTTCCTCAG
	W, reverse	TGTGTATTCCCAAAGTCCCCAG
R26-rox-tdTomato<br>M, 609bp<br>W, 297bp	M, forward	ACGGGTGTTGGGTCGTTTGTTC
	M, reverse	TTCTTGTAATCGGGGATGTCGGCG
	W, forward	AAGGGAGCTGCAGTGGAGTA
	W, reverse	CCGAAAATCTGTGGGAAGTC
R26-RSR-LSL-tdTomato<br>M, 404bp<br>W, 297bp	M, forward	ACGGGTGTTGGGTCGTTTGTTC
	M, reverse	ATGTTTCAGGTTCAGGGGGAGGTG
	W, forward	AAGGGAGCTGCAGTGGAGTA
	W, reverse	CCGAAAATCTGTGGGAAGTC
ACTB-Cre<br>M, 391bp<br>W, 200bp	M, forward	CCTGGAAAATGCTTCTGTCCG
	M, reverse	CAGGGTGTTATAAGCAATCCC
	W, forward	GTATTGAATTGAAGCACCTTTGTTTGG
Ki67-CrexER<br>M, 312bp<br>W, 475bp	reverse	TTGGCGTCTGAAGAGAGTATGACC
	M, forward	GGGCTCTACTTCATCGCATTCC
	W, forward	ATCTGGTTCCCTGGATGGTTG
Ccna2-CrexER<br>M, 677bp<br>W, 726bp	reverse	TGTAACATCTGAGAGCCGAATGAG
	M, forward	GGGCTCTACTTCATCGCATTCC
	W, forward	TGGTAAGAGAAGGGTAAGGGGG
Tnnt2-LSL-mTnG<br>M, 214bp<br>W, 477bp	M, forward	TCCCACAACGAGGACTACACCATC
	M, reverse	CCTTCTTGGAGCCCTTTTTCG
	W, forward	CACACAGTGGAGTCACACAATGG
	W, reverse	ATTATTCTGAGGTCTCGGGGG

M, mutant allele; W, wild type allele.

Southern Blotting probe sequence

ACGTATAGCCGAAATTGCCAGGATCAGGGTTAAAGATATCTCACGTACTGACGG
TGGGAGAATGTTAATCCATATTGGCAGAACGAAAACGCTGGTTAGCACCGCAGG
TGTAGAGAAGGCACTTAGCCTGGGGGTAACTAAACTGGTCGAGCGATGGATTTC
CGTCTCTGGTGTAGCTGATGATCCGAATAACTACCTGTTTTGCCGGGTCAGAAAA

AATGGTGTTGCCGCGCCATCTGCCACCAGCCAGCTATCAACTCGCGCCCTGGAAG
GGATTTTTGAAGCAACTCATCGATTGATTTACGGCGCTAAGGATGACTCTG
