# Supplementary material for: Cell proliferation fate mapping reveals regional cardiomyocyte cell-cycle activity in subendocardial muscle of left ventricle
Source: Nat Commun. 2021 Oct 1;12:5784. doi: 10.1038/s41467-021-25933-5 (PMC8486850; doi:10.1038/s41467-021-25933-5)
Supplement: Supplementary file 2 — Reporting Summary [file 41467_2021_25933_MOESM2_ESM.pdf]

nature portfolio

Corresponding author(s): Bin Zhou

Last updated by author(s): Aug 25, 2021

Reporting Summary

Nature Portfolio wishes to improve the reproducibility of the work that we publish. This form provides structure for consistency and transparency in reporting. For further information on Nature Portfolio policies, see our Editorial Policies and the Editorial Policy Checklist.

Statistics

For all statistical analyses, confirm that the following items are present in the figure legend, table legend, main text, or Methods section.

n/a	Confirmed	
☐	☒	The exact sample size (*n*) for each experimental group/condition, given as a discrete number and unit of measurement
☐	☒	A statement on whether measurements were taken from distinct samples or whether the same sample was measured repeatedly
☐	☒	The statistical test(s) used AND whether they are one- or two-sided<br>*Only common tests should be described solely by name; describe more complex techniques in the Methods section.*
☒	☐	A description of all covariates tested
☒	☐	A description of any assumptions or corrections, such as tests of normality and adjustment for multiple comparisons
☒	☐	A full description of the statistical parameters including central tendency (e.g. means) or other basic estimates (e.g. regression coefficient) AND variation (e.g. standard deviation) or associated estimates of uncertainty (e.g. confidence intervals)
☒	☐	For null hypothesis testing, the test statistic (e.g. *F*, *t*, *r*) with confidence intervals, effect sizes, degrees of freedom and *P* value noted<br>*Give P values as exact values whenever suitable.*
☒	☐	For Bayesian analysis, information on the choice of priors and Markov chain Monte Carlo settings
☒	☐	For hierarchical and complex designs, identification of the appropriate level for tests and full reporting of outcomes
☒	☐	Estimates of effect sizes (e.g. Cohen's *d*, Pearson's *r*), indicating how they were calculated

Our web collection on statistics for biologists contains articles on many of the points above.

Software and code

Policy information about availability of computer code

Data collection	Zeiss stereoscope (Axio Zoom. V16) was used for whole-mount bright-field and fluorescence image collection. Olympus fluorescence microscope (BX53), Nikon A1 confocal, and Olympus FVMPE-RS were used for immunofluorescence data collection. FACS Aria II Flow Cytometer (BD Bioscience) were used for FACS data collection.
Data analysis	Image J (FIJI) and Photoline (18.5.1) were used for immunofluorescence and bright-filed images analysis. FlowJo (Tree star) were used for flow cytometry data analysis. GraphPad Prism 8.0 was used for data analysis.

For manuscripts utilizing custom algorithms or software that are central to the research but not yet described in published literature, software must be made available to editors and reviewers. We strongly encourage code deposition in a community repository (e.g. GitHub). See the Nature Portfolio guidelines for submitting code & software for further information.

Data

Policy information about availability of data

All manuscripts must include a data availability statement. This statement should provide the following information, where applicable:

- Accession codes, unique identifiers, or web links for publicly available datasets
- A description of any restrictions on data availability
- For clinical datasets or third party data, please ensure that the statement adheres to our policy

All data generated by this study are included in this article and its supplemenraty materials. They are available upon request. Source data are provided with this paper

Field-specific reporting

Please select the one below that is the best fit for your research. If you are not sure, read the appropriate sections before making your selection.

- [x] Life sciences
- [ ] Behavioural & social sciences
- [ ] Ecological, evolutionary & environmental sciences

For a reference copy of the document with all sections, see nature.com/documents/nr-reporting-summary-flat.pdf

Life sciences study design

All studies must disclose on these points even when the disclosure is negative.

Sample size	For all experiments, n≥3 was used according to standard scientific conventions, each sample size were described in detail in each figure legend. No statistical methods were used to predetermine the sample size.
Data exclusions	No data were excluded from the analysis
Replication	For each experiment, at least 3 repeats were done to confirm the reproducibility of the findings and all the replication attempts were successful.
Randomization	Mice of both male and female were used at the same age. And they were randomly assigned to different experiment groups.
Blinding	For data collection and analysis, the investigators were not blinded to the group allocation. For injury models, the investigators were blinded for group allocation.

Reporting for specific materials, systems and methods

We require information from authors about some types of materials, experimental systems and methods used in many studies. Here, indicate whether each material, system or method listed is relevant to your study. If you are not sure if a list item applies to your research, read the appropriate section before selecting a response.

Materials & experimental systems

n/a	Involved in the study	
	x	Antibodies
x		Eukaryotic cell lines
x		Palaeontology and archaeology
	x	Animals and other organisms
x		Human research participants
x		Clinical data
x		Dual use research of concern

Methods

n/a	Involved in the study	
x		ChIP-seq
	x	Flow cytometry
x		MRI-based neuroimaging

Antibodies

Antibodies used	Primary antibodies used in immunofluorescence for this study included: ACTN2 (Sigma A7811-.2ML), GFP (Invitrogen A11122), TNNI3 (Abcam AB56357), WGA (Invitrogen W32466), ESR (Abcam Ab27595), tdTomato (Rockland 600-401-379), PECAM (BD Pharmingen 553370), PDGFRa (eBioscience 14-1401-81), E-Cad (R&D AF748), YAP1 (ABclonal A1002), Ki67 (Thermo scientific RM-9106-S0), Nkx2.5 (R&D AF2444). The included secondary antibodies were Alexa donkey anti-mouse 555 (Invitrogen A31570), Alexa donkey anti-rabbit 488 (Invitrogen A21206), Alexa donkey anti-goat 555 (Invitrogen A21432), Alexa donkey anti-goat 647 (Invitrogen A21447), Immpress anti-rabbit immunoglobulin (Vector lab MP-7401-50), TSA Cyanine 3 System (PerkinElmer NEL744A001KT), Alexa donkey anti-rabbit 555 (Invitrogen A31572), Alexa donkey anti-rat 555 (Invitrogen A21434). Primary antibodies used in FACS for this study included: Fc block (eBioscience 14-0161), CD31 APC (eBioscience 17-0311-80), CD140a APC (eBioscience 17-1401-81), CD45 APC (eBioscience 17-0451-82). Hoechst 33342 (beyotime C1022) was included in nucleation analysis. Click-iT cocktail (Invitrogen C10340) was used to stain EdU.
Validation	ACTN2 (Sigma A7811-.2ML), documented to papers with PMID: 33718351, 32109383, 31751568<br>GFP (Invitrogen A11122), documented to papers with PMID: 32311122, 32266943, 32293562<br>TNNI3 (Abcam AB56357), documented to papers with PMID: 33718351, 32109383, 31963369<br>WGA (Invitrogen W32466), documented to papers with PMID: 32220304, 31783035, 17971442<br>ESR (Abcam Ab27595), documented to papers with PMID: 29224780, 29224780, 27516371<br>tdTomato (Rockland 600-401-379), documented to papers with PMID: 33854415, 33833289, 33271069<br>PECAM (BD Pharmingen 553370), documented to papers with PMID: 34168130, 34162553, 34208965<br>PDGFRa (eBioscience 14-1401-81), documented to papers with PMID: 28823869, 27746116, 27325890<br>E-Cad (R&D AF748), documented to papers with PMID: 32796823, 32142663, 30760720<br>YAP1 (ABclonal A1002), documented to papers with PMID: 31883835, 29207260, 31018132

Ki67 (Thermo scientific RM-9106-S0), documented in https://www.fishersci.com/shop/products/ki-67-rabbit-monoclonal-antibody/p-4550778.html
Nkx2.5 (R&D AF2444), documented to papers with PMID: 27806113, 24736402, 24434799
Alexa donkey anti-mouse 555 (Invitrogen A31570), documented to papers with PMID: 28785208, 31053714, 31018127
Alexa donkey anti-goat 647 (Invitrogen A21447), documented to papers with PMID: 31018127, 29907658, 27941801
Immpress anti-rabbit immunoglobulin (Vector lab MP-7401-50), documented to papers with PMID: 32686646, 31988189, 31043676
TSA Cyanine 3 System (PerkinElmer NEL744A001KT), documented to papers with PMID: 28352662, 33550953
Alexa donkey anti-rabbit 555 (Invitrogen A31572), documented to papers with PMID: 31018127, 30416070, 29968757
Alexa donkey anti-rat 555 (Invitrogen A21434), documented to papers with PMID: 27869817, 24859450, 27589684
Fc block (eBioscience 14-0161), documented to papers with PMID: 32931734, 28713239, 31827093
CD31 APC (eBioscience 17-0311-80): documented to papers with PMID: 32094452, 30181538, 29934585
PDGFRa (eBioscience 14-1401-81), documented to papers with PMID: 28823869, 27746116, 27325890
CD45 APC (eBioscience 17-0451-82), documented to papers with PMID: 32702313, 32091393, 30712871
Hoechst 33342 (beyotime C1022), documented to papers with PMID: 31346166, 30772521, 30760623
Click-iT cocktail (Invitrogen C10340), documented to papers with PMID: 23175634, 23015754, 19553203

Animals and other organisms

Policy information about studies involving animals; ARRIVE guidelines recommended for reporting animal research

Laboratory animals	Mice of both male and female at age of 8-10, or 52 weeks were included in this study. All mice were maintained on a 129, C57BL6 and ICR mixed background. All mice were kept in group housing (2-5 mice per cage) in a specific pathogen-free facility with controlled environmental conditions of temperature (20-25°C), humidity (30-70%) and lighting (a 12-h light/dark cycle) at Center for Excellence in Molecular Cell Science, Chinese Academy of Sciences.
Wild animals	No wild animals were included in this study.
Field-collected samples	No field-collected samples were included in this study.
Ethics oversight	All mice were used in accordance with the guidelines of the Institutional Animal Care and Use Committee (IACUC) of Shanghai Institute of Biochemistry and Cell Biology, Chinese Academy of Sciences.

Note that full information on the approval of the study protocol must also be provided in the manuscript.

Flow Cytometry

Plots

Confirm that:

- [x] The axis labels state the marker and fluorochrome used (e.g. CD4-FITC).
- [x] The axis scales are clearly visible. Include numbers along axes only for bottom left plot of group (a 'group' is an analysis of identical markers).
- [x] All plots are contour plots with outliers or pseudocolor plots.
- [x] A numerical value for number of cells or percentage (with statistics) is provided.

Methodology

Sample preparation	Mouse cardiomyocytes were dissociated by digestion buffer (MTS containing 250 U/ml collagenase type 2 (Worthington, LS004176) and 3 U/ml Protease XIV (Sigma, P5147) for about 15 minutes. The digestion buffer were perfused into the heart through the aorta. Then the cell suspension were centrifuged to purify cardiomyocytes by using of Percoll (Sigma, P1644). All the details were included in the methods.
Instrument	Data were collected by FACS Aria II Flow Cytometer (BD Bioscience).
Software	The raw data were processed by FlowJo software (Tree star).
Cell population abundance	About 2x10^5 cardiomyocytes were analyzed for each sample. Cardiomyoctes were determined by the forward scatter and side scatter. For further purifiction, cell suspension were stained with antibodies against endothelial cells (CD31-APC), fibroblasts (CD140a-APC), and hematopoietic cells (CD45-APC) to allow exclusion of these cells.
Gating strategy	The starting cell population were gated from the top right corner in the first gate (FSC/SSC) because cardiomyocytes were larger than many other cell types. The boundaries for BV421 and FITC were determined by comparison with the blank group. The boundary for lineage negative cells was determined by comparison with isotype controls stained group.

- [x] Tick this box to confirm that a figure exemplifying the gating strategy is provided in the Supplementary Information.
