# Supplementary figures and images for: Cell proliferation fate mapping reveals regional cardiomyocyte cell-cycle activity in subendocardial muscle of left ventricle
Source: Nat Commun. 2021 Oct 1;12:5784. doi: 10.1038/s41467-021-25933-5 (PMC8486850; doi:10.1038/s41467-021-25933-5)

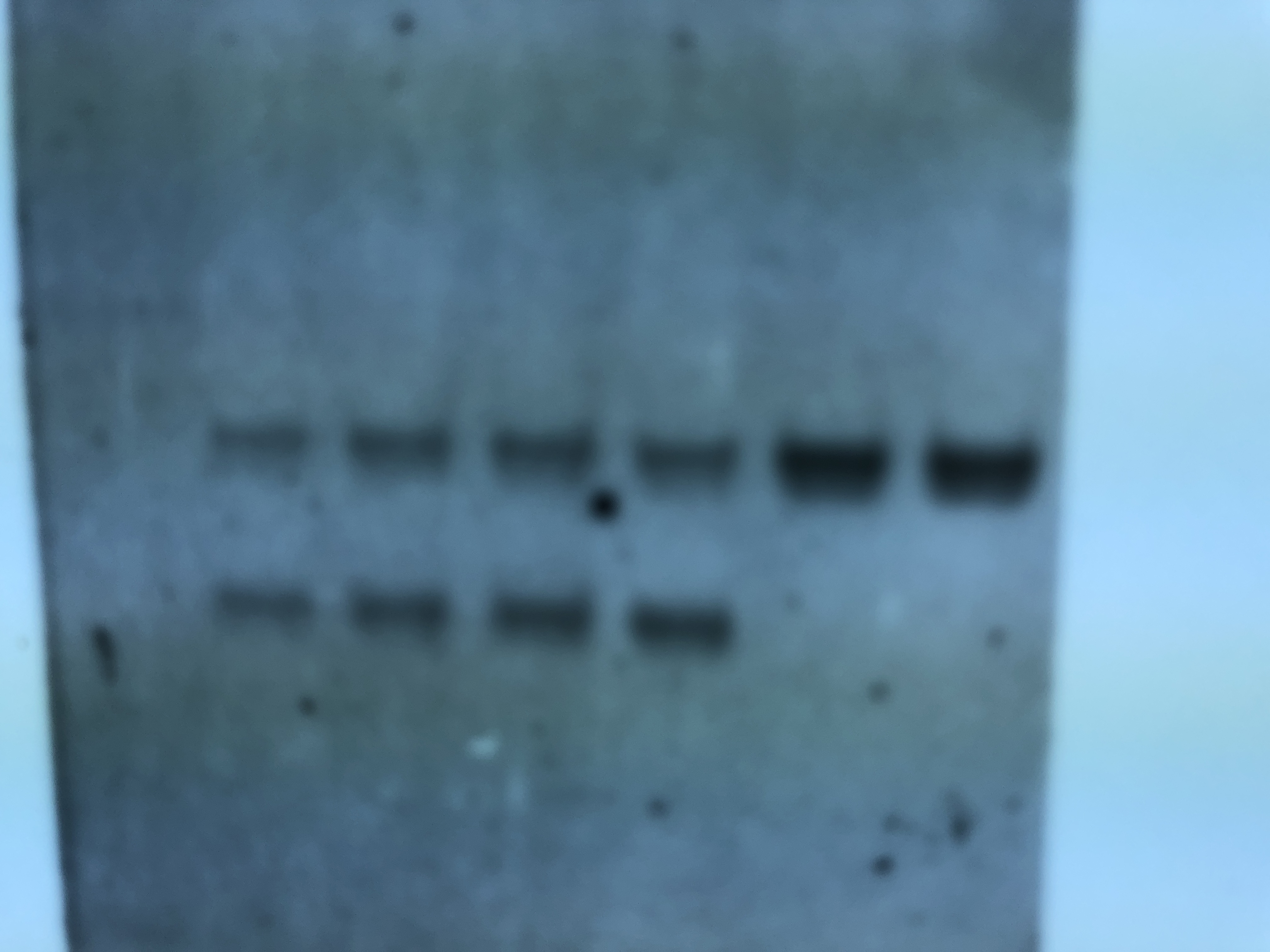

Supplement: Supplementary file 3 — Source Data [file 41467_2021_25933_MOESM3_ESM.zip › 285761_2_supp_5842847_qyclf9.jpg]
